# Pathogens on High-Touch Surfaces in an Arid Megacity: A Longitudinal Molecular Surveillance Study

**DOI:** 10.3390/microorganisms14030626

**Published:** 2026-03-10

**Authors:** Mohamad Taisir Ahmad Ghiba, Saleh Ahmed Eifan, Abdulkarim Fahad Alhetheel, Atif Hanif

**Affiliations:** 1 Botany and Microbiology Department, College of Science, King Saud University, Riyadh 11451, Saudi Arabia; 443105998@student.ksu.edu.sa (M.T.A.G.);; 2 Department of Infection and Immunity, King Faisal Specialist Hospital and Research Center, Riyadh 11211, Saudi Arabia; 3 Department of Pathology and Laboratory Medicine, College of Medicine, King Saud University, Riyadh 11451, Saudi Arabia

**Keywords:** environmental surveillance, fomites, Riyadh, SARS-CoV-2, *Cryptosporidium*, indoor air quality, arid climate

## Abstract

Contaminated environmental surfaces (fomites) act as pathogen reservoirs, yet surveillance data in arid megacities like Riyadh, Saudi Arabia—characterized by extreme heat and indoor climate control—remain limited. This study established a city-wide molecular baseline for surface contamination and evaluated meteorological influences. We conducted a stratified, longitudinal study (February 2023–May 2024), collecting 270 swabs from seven zones, including hospitals, airports, ATMs, and community hubs. Samples were pooled (N = 55) and screened using QIAstat-Dx multiplex PCR panels. Nineteen pools (34.5%) tested positive. Viral pathogens (SARS-CoV-2, Adenovirus, Rhinovirus) were detected in 10 pools (18.2%) and non-viral pathogens (bacteria/parasites) in 13 pools (23.6%), with 7.3% co-detections. Hospitals and airports emerged as primary hubs for respiratory viruses, while *Cryptosporidium* was the most frequent non-viral pathogen (*n* = 6), predominating on ATM interfaces. Binary logistic regression indicated that higher ambient temperature was significantly associated with detecting viral rather than non-viral pathogens among positive samples (OR = 1.728, *p* = 0.032). Despite outdoor aridity, public surfaces in Riyadh harbored diverse pathogens. The link between heat and viral detection suggests indoor microclimates drive persistence during hot seasons, necessitating targeted hygiene measures in high-risk nodes.

## 1. Introduction

Environmental reservoirs complicate the management of infectious diseases globally. Respiratory and gastrointestinal pathogens, including SARS-CoV-2, influenza, and norovirus, move through contaminated surfaces (fomites) and create opportunities for indirect spread [1,2]. While the field has debated exactly how much fomites contribute compared with airborne transmission—especially during the COVID-19 pandemic—evidence indicates that high-touch surfaces in dense urban settings meaningfully support pathogen dissemination [3,4,5]. Most of our current understanding of environmental persistence comes from temperate regions in North America, Europe, and East Asia. In those settings, seasonal viral peaks typically align with colder temperatures and lower humidity, conditions known to enhance the stability of enveloped viruses [6,7]. This “temperate bias” left a practical gap in the literature: we knew far less about how pathogens behave across the hot, arid climates typical of the Middle East and the Gulf Cooperation Council (GCC) countries.

Riyadh, Saudi Arabia, offers a clear test case. Summer ambient temperatures frequently exceed 45 °C, while relative humidity often drops below 15% [8,9]. Classical environmental virology suggests that such conditions should speed the inactivation of enveloped viruses and reduce fomite-related risks [10,11]. However, that “outdoor sterilization” hypothesis did not reflect how modern arid cities function day to day. Riyadh operates as an “indoor city,” where routine life occurs almost exclusively in air-conditioned malls, offices, schools, and homes held at 20–24 °C [12,13,14,15]. We hypothesized that these indoor microclimates might shield pathogens from the harsh outdoor environment, potentially supporting year-round persistence [16,17]. Riyadh also serves as a major transit gateway for millions of religious pilgrims traveling for Hajj and Umrah [18]. This scale of movement creates repeated opportunities for pathogen introduction, as illustrated by earlier MERS-CoV and SARS-CoV-2 events [19]. However, comprehensive molecular baselines for surface contamination in Riyadh remained elusive. Prior studies primarily focused on clinical areas or specific single pathogens, leaving the broader environmental burden unclear [20].

We designed this study to address the “arid urban gap” through a longitudinal, multi-site molecular surveillance campaign. We aimed to: (1) measure the prevalence and diversity of respiratory and gastrointestinal pathogens on high-touch surfaces in public spaces; (2) identify specific contamination hotspots; and (3) investigate the relationship between meteorological factors (temperature and humidity) and the differential detection of viral versus non-viral pathogens. Our principal findings reveal that, contrary to the outdoor sterilization hypothesis, higher ambient temperatures were associated with increased viral detection, suggesting that indoor behavioral dynamics drive pathogen persistence in arid megacities.

## 2. Materials and Methods

### 2.1. Study Design and Setting

**Figure 1:**
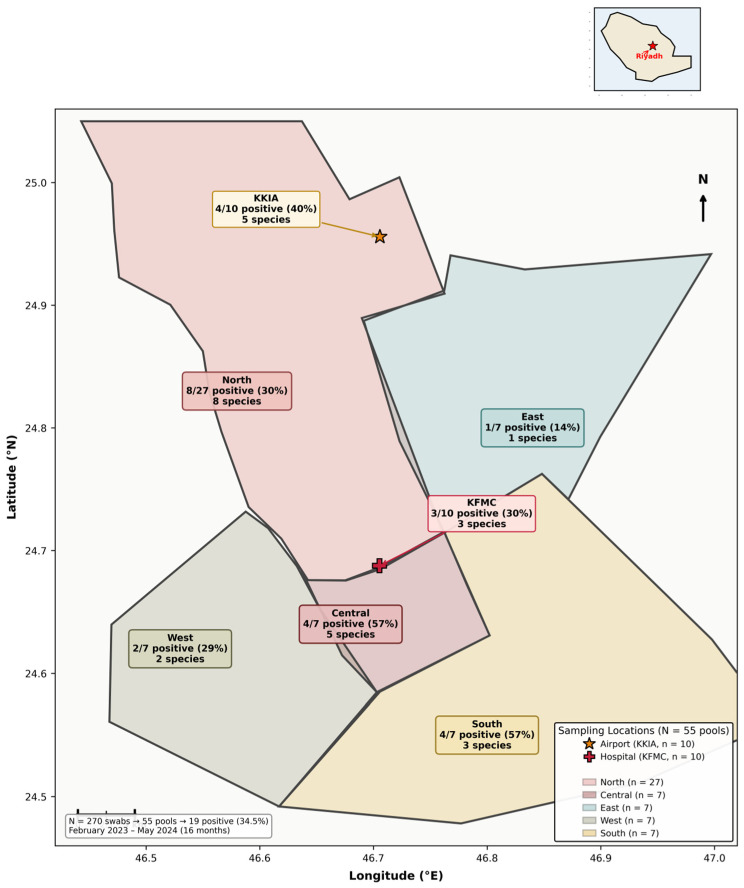
Geographical distribution of environmental sampling zones across Riyadh city districts. Five community regions (North, Central, East, West, South) and two institutional hubs (King Khalid International Airport [KKIA] and King Fahad Medical City [KFMC]) were surveyed over 16 months (February 2023–May 2024). N = 270 swabs aggregated into 55 pools; 19 positive (34.5%). Coordinate reference system: WGS 84 (EPSG:4326).

We conducted a stratified, longitudinal environmental surveillance study over 16 months, from February 2023 to May 2024. The work took place across the Riyadh metropolitan area, which we divided into seven geographic zones to ensure representative coverage: five community regions (North, South, East, West, and Central Riyadh) and two high-significance institutional hubs—King Fahad Medical City (KFMC; 24.6885° N, 46.7028° E) and King Khalid International Airport (KKIA; 24.9576° N, 46.6988° E). The five community sampling zones and two institutional facilities are shown in Figure 1.

The five community regions were: North Riyadh (24.736° N, 46.682° E), South Riyadh (24.584° N, 46.774° E), East Riyadh (24.695° N, 46.752° E), West Riyadh (24.633° N, 46.535° E), and Central Riyadh (24.666° N, 46.711° E); multiple sites within each region were sampled (Figure 1). All sampling was anchored within the Riyadh metropolitan area (centered on approximately 24.7136° N, 46.6753° E). Stratified spatial designs are widely used to improve cost-efficiency and geographic representativeness in environmental surveys [21].

### 2.2. Sampling Strategy and Site Selection

We selected sites after identifying the busiest touchpoints in each setting to maximize the chance of detecting contamination [22,23]. Prior studies indicate that high-touch non-porous surfaces in community settings can harbor respiratory viral RNA and correlate with local COVID-19 trends [24]. We collected a total of 270 environmental swabs (Table 1). Target surface types included the following:

- Healthcare Facilities (KFMC): Waiting area armchairs, reception counters, clinic door handles, and restroom sinks. These surfaces represented routine interfaces among patients, healthcare workers, and visitors [25].
- Transportation Hubs (KKIA): Check-in counters, luggage trolleys, escalator handrails, food court tables, and restroom facilities. Travelers repeatedly handled these surfaces at peak flow times.
- Community Hubs: Public parks (benches, play equipment), mosques (ablution areas, door handles), supermarkets (shopping cart handles, payment terminals), and coffee shops (tables, counters).
- Financial Services: Automated Teller Machines (ATMs), stratified into indoor (bank branch) and outdoor (drive-through or street-side kiosk) placements.

### 2.3. Sample Collection Protocol

To improve recovery from dry, non-porous surfaces, we used sterile flocked nylon swabs (SAMCO MDM 750, Second Advance Medical Company, Riyadh, Saudi Arabia) pre-moistened with 2 mL of Viral Transport Medium (VTM) [26,27]. Flocked nylon swabs significantly enhance microbial and viral recovery from environmental and clinical surfaces compared with traditional fiber swabs, including under dry-surface conditions [25,28,29,30]. For each sample, we swabbed approximately 100 cm^2^ using a consistent grid approach (vertical, horizontal, and diagonal strokes) while rotating the swab to maximize uptake. For smaller or irregular targets, such as door handles or ATM keypads, we swabbed the entire functional contact area.

We managed strict aseptic protocols to prevent cross-contamination. Personnel wore PPE, including gloves and masks, and changed gloves between every sample [31]. We placed samples into sterile transport tubes immediately, kept them on ice (4 °C) during transport, and ensured transfer to −80 °C storage to maintain viral RNA stability [32].

### 2.4. Sample Pooling and Extraction

We combined the 270 individual swabs into 55 pooled samples. We pooled based on spatial and temporal logic; each pool contained 4–5 swabs taken from the same location type on the same date. This pooling approach has been widely used in infectious disease surveillance [33,34] and can improve efficiency and reduce costs for SARS-CoV-2 RT-PCR screening when pool sizes remain modest [35,36,37].

We performed nucleic acid extraction using the QIAstat-Dx automated cartridge system, which integrated lysis, purification, and elution. Automated cartridge-based nucleic acid platforms provide robust workflows for diverse clinical and environmental specimens [38]. Each cartridge included an internal control (MS2 bacteriophage) to allow us to detect inhibition from dust or cleaning agents common in environmental matrices.

The composition of the pooled samples is summarized in Table 2 (Results).

### 2.5. Molecular Detection (Multiplex PCR)

We screened samples using the QIAstat-Dx Respiratory SARS-CoV-2 Panel and the QIAstat-Dx Gastrointestinal Panel 2 (QIAGEN, Hilden, Germany). These syndromic assays used real-time reverse-transcription PCR (RT-PCR) to detect multiple targets in parallel. Independent evaluations of the QIAstat-Dx Respiratory SARS-CoV-2 Panel reported good agreement with reference RT-PCR workflows for several respiratory targets [39,40].

- Respiratory Panel Targets: SARS-CoV-2, Influenza A/B, RSV, Adenovirus, Rhinovirus/Enterovirus, Parainfluenza (1–4), Human Metapneumovirus, *Bordetella pertussis*, *Legionella pneumophila*, and *Mycoplasma pneumoniae*.
- Gastrointestinal Panel 2 Targets: Norovirus (GI/GII), Rotavirus A, Adenovirus F40/41, *Salmonella* spp., *Campylobacter* spp., *Vibrio cholerae*, *C. difficile*, ETEC, EPEC, EAEC, *Cryptosporidium*, *Giardia lamblia*, and *Entamoeba histolytica* [41].

### 2.6. Statistical Analysis

All statistical analyses were conducted using R (version 4.5.2; R Core Team, 2025) via RStudio (version 2025.09.2+418). Descriptive summaries captured the overall prevalence and the distribution of detected pathogens. The main outcome variable was detection of any pathogen (binary: positive/negative). For pools that tested positive, a second outcome classified pathogen type (Viral vs. Non-Viral).

Important note on classification: For the binary logistic regression, we grouped the four pools with co-detection (Viral + Non-Viral) into the “Viral” category to focus the model on respiratory viral drivers. As a result, inferential comparisons tested “Viral/Mixed” (*n* = 10) versus “Non-Viral Only” (*n* = 9). The descriptive results, however, reported total non-viral detections (*n* = 13 pools with non-viral detections) to reflect the full set of organisms detected.

We recorded ambient temperature (°C) and humidity at sampling time. Shapiro–Wilk testing indicated non-normal distributions for these meteorological variables, so we used non-parametric comparisons (Mann–Whitney U and Kruskal–Wallis tests) [42]. To test whether meteorological conditions are related to pathogen composition, we constructed a binary logistic regression model using Pathogen Type as the outcome and temperature and humidity as predictors. We assessed model performance using overall model significance, Nagelkerke R^2^ [43], and classification accuracy; the Hosmer–Lemeshow test could not be computed due to the small sample size. Statistical significance was set at *p* < 0.05.

## 3. Results

A total of 270 environmental surface swabs were collected and aggregated into 55 pooled samples for molecular analysis. The study covered five distinct setting categories across Riyadh, including community hubs, institutional centers, and financial service points. Overall, pathogen DNA or RNA was detected in 19 of the 55 pools, corresponding to a cumulative prevalence of 34.5%. The distribution of sampling effort and positivity rates across these settings is summarized in Table 2.

The complete pool-level metadata, including constituent swab identifiers, collection dates, surface types, and environmental conditions for all 55 pools, is provided in Supplementary Table S1.

### 3.1. Overall Prevalence and Pathogen Diversity

The analysis included 55 pooled samples representing 270 individual swabs. Nineteen pools tested positive for at least one target pathogen, producing an overall positivity rate of 34.5%. The remaining 36 pools (65.5%) tested negative. Positive pools contained a broad mix of viral, bacterial, and parasitic targets:

- Viral Pathogens: Detected in 10 pools (18.2% of total). The most frequent viral detections were SARS-CoV-2 (4 pools), Rhinovirus/Enterovirus (3 pools), and Adenovirus (3 pools).
- Non-Viral Pathogens: Detected in 13 pools (23.6% of total). This category included bacteria (*Vibrio cholerae*, *Salmonella*, pathogenic *E. coli*) and parasites (*Cryptosporidium*).

### 3.2. Co-Detection Patterns

Four pools (7.3%) showed co-detection of viral and non-viral pathogens:

- Pool CP-17: Co-detection of SARS-CoV-2 and *Vibrio cholerae*.
- Pool CP-20: Multi-pathogen detection of SARS-CoV-2, *Cryptosporidium*, and Enteroaggregative *E. coli* (EAEC).
- Pool HP-10: Co-detection of Adenovirus and EAEC.
- Pool AP-7: Co-detection of Coronavirus 229E and *Cryptosporidium*.

**Figure 2:**
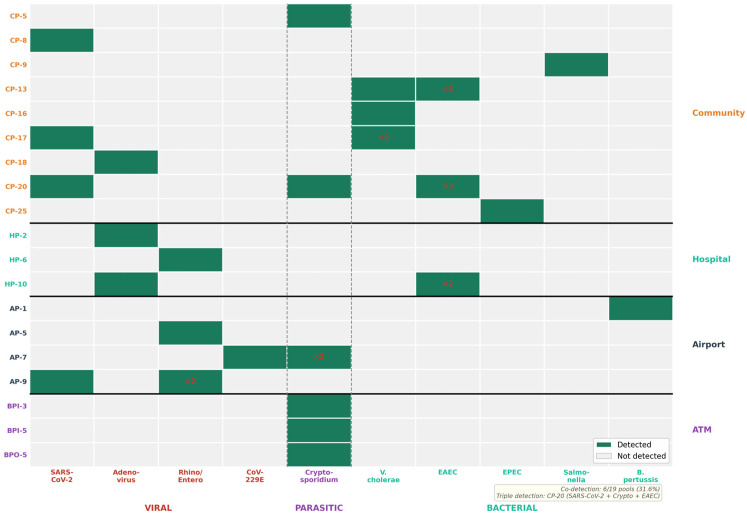
Pathogen co-detection heatmap across 19 positive pools. Presence/absence matrix of 10 pathogens grouped by site category, with co-detection events highlighted.

Additionally, two pools showed within-category multi-pathogen co-detection: Pool CP-13 contained Vibrio cholerae and EAEC (dual enteric contamination), and Pool AP-9 contained Rhinovirus/Enterovirus and SARS-CoV-2 (dual respiratory viral contamination). The complete co-detection matrix is visualized in Figure 2. In total, 6 of 19 positive pools (31.6%) harbored ≥2 distinct pathogens.

To assess whether multi-pathogen co-detection reflected non-random contamination clustering, we compared observed co-detection rates to those expected under an independence model based on marginal species prevalences. Among 19 positive pools, 6 (31.6%) harbored ≥2 pathogens, compared to an expected rate of 19.8% under independence (expected: 3.76 pools; observed: 6 pools; ratio: 1.6×). While this excess did not reach statistical significance (binomial test, *p* = 0.156), the systematic co-occurrence of respiratory viruses with enteric pathogens on restroom surfaces suggests shared contamination pathways rather than independent deposition events.

### 3.3. Detailed Pathogen Counts

From the raw detection outputs, the observed frequencies were:

- *Cryptosporidium* spp.: Detected most often, appearing in 6 pools.
- SARS-CoV-2: Detected in 4 pools.
- *Vibrio cholerae*: Detected in 3 pools.
- Pathogenic *E. coli*: EAEC (*n* = 3) and EPEC (*n* = 1).
- Adenovirus: Detected in 3 pools.
- Rhinovirus/Enterovirus: Detected in 3 pools.
- Other detections: *Salmonella* (*n* = 1), *Bordetella pertussis* (*n* = 1), Coronavirus 229E (*n* = 1).

The complete pathogen-specific detection profile, including Ct values, site associations, plausible circulation pathways, and recommended control measures, is presented in Table 3.

### 3.4. Site-Specific Contamination Profiles

**Figure 3:**
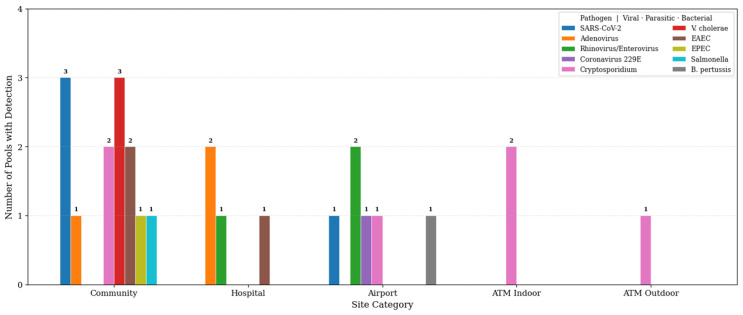
Distribution of specific pathogens across site categories (Community, Hospital, Airport, ATM Indoor, and ATM Outdoor) at the species/genus level.

**Figure 4:**
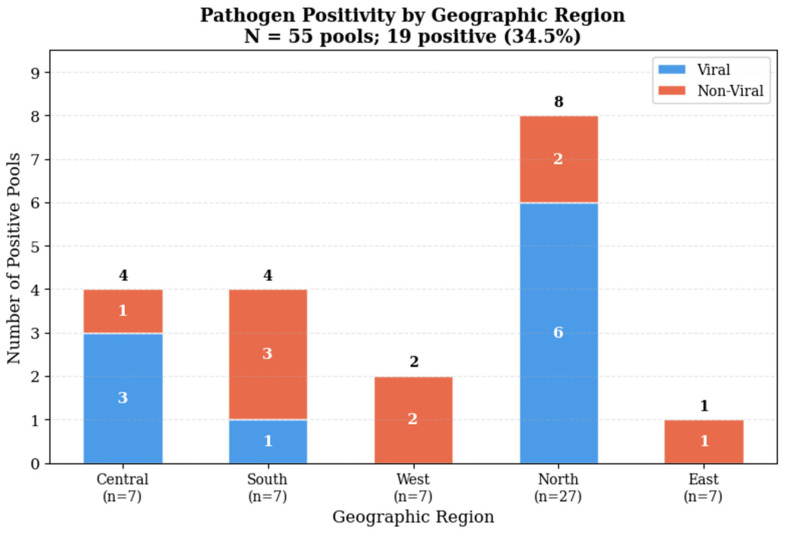
Pathogen positivity by geographic region and site type. Stacked bars showing the number of positive pools per Riyadh zone, stratified by viral and non-viral detections. N = 55 pools; 19 positive.

Pathogen distribution varied across zones and showed clear site-linked patterns (Figure 3 and Figure 4).

- Community Hubs (Parks, Cafes, Markets): This zone carried the highest absolute burden, with 9 positive pools (36% positivity). The profile was diverse, including SARS-CoV-2, *Salmonella*, *Vibrio cholerae*, and *Cryptosporidium*. The mix likely reflected high turnover, variable hygiene practices, and broad public access.
- Airports (KKIA): Four pools tested positive (40% positivity). Detections included SARS-CoV-2, Rhinovirus, and Coronavirus 229E, consistent with respiratory introduction through travel. One airport pool also contained *Bordetella pertussis*, and another contained *Cryptosporidium*.
- Hospitals (KFMC): Three pools tested positive (30% positivity). The profile leaned toward respiratory viruses (Adenovirus, Rhinovirus) and enteric bacteria (EAEC). Adenovirus appeared on a hospital toilet door handle and waiting area seating, highlighting routine infection-control pressure points in high-traffic areas.
- Banking Services (ATMs): Three pools tested positive. A clear pattern emerged: positive ATM pools in this zone yielded only *Cryptosporidium*, with no viruses or bacteria co-detected. This suggested that ATM keypads functioned as a selective niche where hardy *Cryptosporidium* oocysts persisted, while more fragile viral envelopes and bacterial cells likely degraded under desiccation and heat.

### 3.5. Surface Contamination Risk Ranking

**Figure 5:**
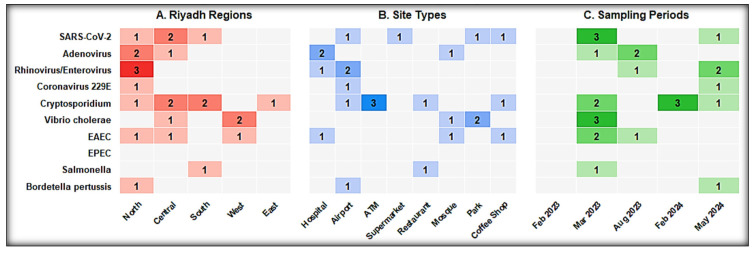
Cross-tabulation heatmaps of pathogen detection by site type, surface category, and sampling period. Three heatmaps displaying the number of positive pool detections for each of 10 identified pathogens across (**A**) five Riyadh regions, (**B**) eight site types, and (**C**) five sampling periods. Cell values indicate the number of pools in which the pathogen was detected. Color intensity corresponds to detection frequency; darker shading indicates a higher number of detected pools. N = 55 pools; 19 positive.

To translate molecular detection data into actionable risk stratification, surface types were ranked by positivity rate, pathogen diversity, and co-detection frequency (Table 4). Extended cross-tabulations of pathogen detection by site type, surface category, and sampling period are presented in Figure 5.

Restroom surfaces ranked highest across all metrics: highest positivity (44.4%), highest diversity (all 10 species detected), and highest co-detection rate (42%), identifying restrooms as the primary convergence zone for multi-route pathogen transmission. ATM touchscreens (indoor) ranked second in terms of positivity (40.0%) but showed *Cryptosporidium* exclusively with zero co-detection, indicating a single-pathway contamination mechanism requiring oocysticidal disinfection. Hospital surfaces showed moderate positivity (30.0%) with intermediate diversity and 33% co-detection, reflecting healthcare-associated respiratory and enteric pathogen overlap. Outdoor ATM surfaces ranked lowest (20.0%), suggesting that UV exposure and environmental weathering reduce but do not eliminate parasitic contamination.

The surface risk ranking informed the recommended disinfection strategy by surface type (Table 5).

**Figure 6:**
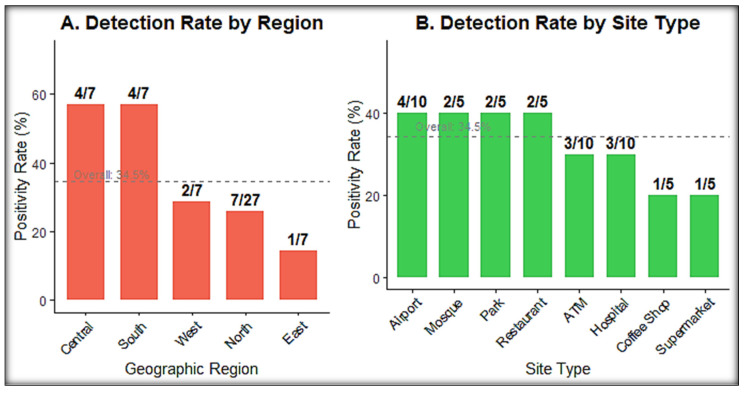
Pathogen detection rates across geographic regions and site types. Two-panel bar chart showing positivity rates (%) with (**A**) five geographic regions (Central, South, West, North, East) and (**B**) eight site types (Hospital, Airport, ATM, Supermarket, Restaurant, Mosque, Park, Coffee Shop). Counts shown as (positive/total) beneath each bar. N = 55 pools; 19 positive (34.5%).

**Figure 7:**
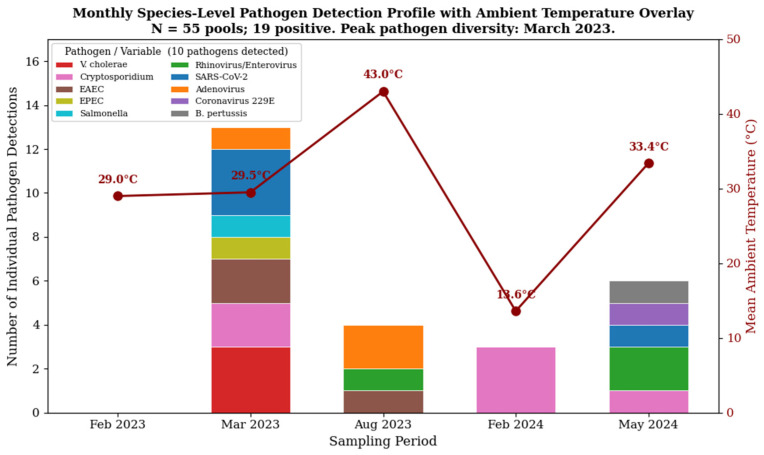
Monthly species-level pathogen detection profile with ambient temperature overlay. Stacked bar chart showing the number of individual pathogen detections per sampling period (February 2023, March 2023, August 2023, February 2024, May 2024), colored by pathogen identity (10 pathogens; see legend). Red line with markers: mean ambient temperature (°C). Peak pathogen diversity occurred in March 2023. N = 55 pools; 19 positive.

The detection of infectious agents exhibited a distinct temporal trend during the study period. As illustrated in Figure 6 (species-level monthly profiles are shown in Figure 7), viral pathogens were predominantly detected during the warmer summer months (August), whereas bacterial and parasitic agents were more frequently identified during the cooler winter/spring season (February–March). This seasonal divergence was not statistically significant (χ^2^ = 1.35, *p* = 0.246), suggesting that environmental conditions in Riyadh—specifically the extreme summer heat driving populations indoors—may differentially select for viral persistence on high-touch surfaces.

### 3.6. Environmental Drivers: The Temperature Effect

To examine the “Indoor City” hypothesis, we analyzed the relationship between meteorological variables and pathogen detection in three stages.

#### 3.6.1. Comparison Across All Groups

**Figure 8:**
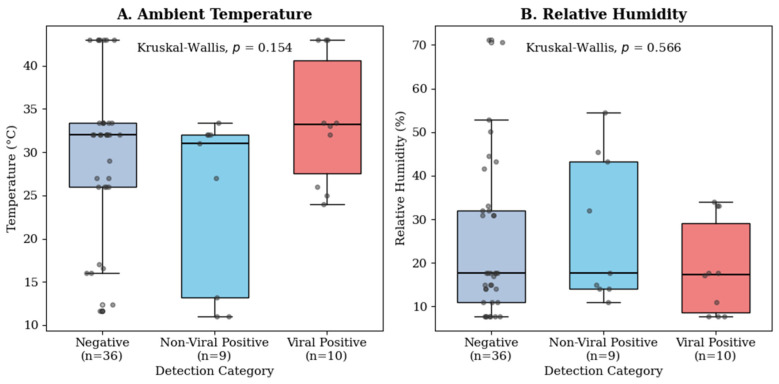
Environmental conditions by pathogen detection category. Boxplots comparing (**A**) ambient temperature (°C) and (**B**) relative humidity (%) distributions across Negative (*n* = 36), Non-Viral Positive (*n* = 9), and Viral Positive (*n* = 10) pools. Kruskal–Wallis H tests shown in each panel. No statistically significant differences were observed (α = 0.05). N = 55 pools; 19 positives.

First, a Kruskal–Wallis H test was conducted to compare median temperatures across the three outcome groups: Negative, Non-Viral Positive, and Viral Positive. While the Viral Positive group had the highest median temperature (33.2 °C), the overall difference across groups was not statistically significant (H(2) = 3.74, *p* = 0.154) (Figure 8). Air quality index distributions across pathogen detection categories are presented in Figure S2.

To isolate the specific effect of environmental conditions on pathogen survival, we constructed a binary logistic regression model comparing Viral Positive pools against Non-Viral and Negative pools. The analysis identified ambient temperature as a significant positive predictor of viral detection. Specifically, for every 1 °C increase in temperature, the odds of detecting a viral pathogen increased significantly, reinforcing the observation that viral targets were more frequently recovered during the warmer sampling periods. The magnitude of this environmental effect is detailed in Table 6.

#### 3.6.2. Viral Positive vs. Negative Pools

Second, we compared pools positive for viruses (*n* = 10) against negative pools (*n* = 45). Although viral-positive pools were associated with a slightly higher median temperature (33.2 °C) than negative pools (32.0 °C), this difference was not statistically significant (*p* = 0.225). Binary logistic regression confirmed that neither temperature nor humidity significantly predicted the presence of a virus compared to a negative result (Overall model: χ^2^(2) = 2.578, *p* = 0.267).

#### 3.6.3. Viral vs. Non-Viral Positive Pools

Finally, to test whether meteorological conditions could differentiate between types of contamination, we performed a binary logistic regression comparing only the positive pools (N = 19) (Table 7). In this model, higher ambient temperature was a significant predictor of viral detection (χ^2^(2) = 9.345, *p* = 0.009; Nagelkerke R^2^ = 0.518).

- Temperature: Higher ambient temperature significantly predicted viral detection (OR = 1.728, 95% CI [1.050, 2.845], *p* = 0.032). For each 1 °C increase, the odds that a positive sample contained a virus (rather than a non-viral pathogen) rose by 72.8%.
- Humidity: Relative humidity did not reach significance in this model (*p* = 0.200).

#### 3.6.4. UV Index as Environmental Correlate

UV index data (categorical: Low, Moderate, High, Very High) were available for all 55 pools. Among the 19 positive pools, a distinct pattern emerged: at Low UV index, all five detections (100%) were non-viral pathogens (*Cryptosporidium* and enteric bacteria), whereas 10 of 11 viral-positive pools were detected at High or Very High UV levels. Fisher’s exact test confirmed that viral detection was significantly associated with higher UV categories (Low + Moderate: 1/22 pools viral; High + Very High: 10/33 pools viral; OR = 0.12, *p* = 0.036). This pattern parallels the temperature finding: high UV co-occurs with extreme heat, and both drive populations indoors, indirectly increasing respiratory virus deposition on climate-controlled indoor surfaces (Figure S6).

ROC Analysis: ROC analysis confirmed temperature as a statistically significant classifier of viral versus non-viral positive pools (AUC = 0.756, 95% CI [0.532, 0.979], *p* = 0.025), with an optimal cutoff of >32.50 °C yielding 60.0% sensitivity and 88.9% specificity.

Additional supplementary visualizations are provided: functional surface zone analysis (Figure S1), AQI and detection (Figure S2), multivariate environmental analysis (Figure S3), AQI versus pathogen load (Figure S4), pathogen diversity with co-detection patterns (Figure S5), UV index analysis (Figure S6), and environmental trend analysis (Figure S7). Complete pool-level metadata is provided in Supplementary Table S1.

## 4. Discussion

This study provided a broad molecular snapshot of high-touch surface contamination across Riyadh and established a baseline for environmental pathogen burden in an arid megacity, consistent with work showing that high-touch “hubs” can act as key nodes for microbial dissemination in public spaces [3,44]. The overall positivity rate of 34.5% matched, and in some cases exceeded, rates reported from temperate hospitals where ~63% of high-touch sites surpassed accepted cleanliness thresholds and a substantial fraction showed moderate–heavy contamination [45,46]. This reinforces evidence that frequently touched surfaces in community and healthcare environments often harbor diverse microorganisms at moderate-to-high loads [47,48]. That result pushed back against the assumption that the regional climate alone ensured environmental sterility.

### 4.1. The “Indoor City” Paradox: Heat and Viral Persistence

The most striking signal was the positive association between ambient temperature and viral detection (OR = 1.728). Experimental work has consistently shown that heat speeds inactivation of enveloped viruses such as SARS-CoV-2 [10] and other coronaviruses, with faster decay at higher temperatures on surfaces [49,50]. In a purely outdoor model, viral recovery should have dropped as temperatures climbed toward 45 °C.

The observed pattern pointed in a different direction. In Riyadh, higher outdoor heat likely shifted daily behavior. People moved indoors and stayed there longer, relying on climate-controlled spaces maintained around 20–24 °C with low humidity. Those indoor conditions could favor viral stability and increase airborne and fomite exposure. While this study did not measure ventilation directly, the literature links poor indoor air exchange to prolonged survival of respiratory viruses [51,52,53]. In that sense, “high temperature” in the dataset behaved like a proxy for “high indoor density.” More people indoors meant more shedding and more surface contact in malls, terminals, and hospitals, aligning with models suggesting that dense networks of high-touch surfaces can accelerate the redistribution of contamination [44]. That indoor load plausibly outweighed any outdoor “sterilization” effect from the sun and heat.

The temperature–detection association observed in this study is consistent with established evidence on the environmental determinants of pathogen RNA persistence. SARS-CoV-2 nucleic acid demonstrates greater stability in cold, dry environments, with surface detectability measured in days at 4 °C but declining to hours at temperatures exceeding 37 °C [54]. At temperatures above 38 °C—common outdoors in Riyadh for approximately five months of the year—thermal degradation acts as a natural reduction mechanism for enveloped viral RNA on exposed surfaces [55]. However, this outdoor effect is structurally bypassed in Riyadh’s built environment, where indoor temperatures are maintained at 20–24 °C by ubiquitous air conditioning—within the commonly reported range for respiratory virus transmission (15–25 °C) [6,15].

Humidity adds a second layer of complexity to the Indoor City framework. The relationship between relative humidity and viral RNA persistence follows a U-shaped curve: many respiratory viruses remain detectable longer in very dry (<40% RH) or very humid (>75% RH) environments, with more rapid nucleic acid degradation occurring at intermediate relative humidity (50–70% RH) [56]. Riyadh’s outdoor humidity frequently falls below 15% RH, while indoor air-conditioned spaces typically maintain 30–50% RH—both within the low-humidity persistence zone. This means that neither the outdoor nor the indoor humidity regime in Riyadh’s arid environment reaches the intermediate zone that would accelerate viral RNA decay, potentially contributing to the detection of enveloped viral nucleic acid on both indoor and outdoor surfaces in our dataset.

UV radiation represents the third environmental variable with relevance to surface pathogen dynamics. High-intensity UV radiation from sunlight accelerates degradation of SARS-CoV-2 and other enveloped viral RNA on surfaces, significantly reducing their outdoor detectability compared to shaded indoor environments [57,58]. Our exploratory analysis of UV index as a correlate of detection type (Section 3.6.4) revealed a significant association: viral detections were concentrated at High and Very High UV index categories (Fisher’s exact *p* = 0.036), while all detections at Low UV were exclusively non-viral. Importantly, this does not imply that UV increases viral persistence; rather, high UV co-occurs with extreme heat, and both together drive populations indoors, increasing respiratory virus deposition on climate-controlled indoor surfaces. UV index thus acts as an indirect proxy for indoor congregation behavior rather than a direct predictor of viral persistence.

Together, these three environmental variables—temperature, humidity, and UV radiation—operate in concert to shape the distinct contamination patterns observed across Riyadh’s indoor and outdoor surfaces. While mechanistic studies describe viability changes under controlled conditions, our dataset assesses nucleic acid detection rather than infectious viability; the environmental associations reported here should therefore be interpreted as correlates of RNA detectability patterns rather than direct measures of infection risk.

### 4.2. Pathogen-Specific Ecological Signals and Plausible Circulation Pathways

While the viral/non-viral binary classification served the specific statistical purpose of testing whether climatic conditions differentially predict pathogen type, the QIAstat-Dx panels identified pathogens at the species or genus level, revealing distinct ecological signatures across site types. The following sections interpret each major detection in the context of established transmission mechanisms and the arid indoor-city environment. All interpretations represent plausible pathways inferred from the consistency of site–pathogen associations with published mechanistic evidence; source attribution cannot be established from observational pooled surface detections.

SARS-CoV-2 (4 pools: CP-8, CP-17, CP-20, AP-9)

SARS-CoV-2 was the most frequently detected viral pathogen, recovered from community surfaces (supermarket carts, park benches, coffee shop tables) and an airport food court table. The relatively low Ct values (33.9–34.8), while above thresholds typically associated with recoverable infectious virus in clinical samples, suggest more recent contamination than the high-Ct (>35) detections of other pathogens in this dataset, though Ct values alone cannot establish viability. Detection is consistent with respiratory droplet deposition and direct hand contact from symptomatic or pre-symptomatic individuals. In air-conditioned indoor environments maintained at 20–24 °C, HVAC systems may contribute to redistribution of aerosolized virus to surfaces distant from the source individual, expanding the spatial footprint of fomite contamination [59]. The co-detection with *V. cholerae* in CP-17 and with *Cryptosporidium* and EAEC in CP-20 is consistent with convergent multi-source contamination at high-traffic community surfaces, where respiratory, fecal–oral, and hand-to-surface transmission routes overlap.

The outdoor half-life of SARS-CoV-2 at temperatures exceeding 27 °C is less than 2 h, and UV irradiance at Riyadh’s summer levels (>900 W/m^2^) further accelerates inactivation on unshaded surfaces [55]. The detection of SARS-CoV-2 during the warm sampling period (August 2023) therefore reflects the Indoor City dynamic: virus deposited on indoor surfaces at air-conditioned temperatures persists in conditions where outdoor surfaces experience rapid inactivation of enveloped viruses. This finding is consistent with the framework’s prediction that indoor high-touch surfaces in arid megacities serve as recurring sites of enveloped respiratory virus contamination even during extreme summer heat.

Adenovirus (3 pools: CP-18, HP-2, HP-10)

Adenovirus was detected on a mosque door handle (CP-18), a hospital waiting area seat (HP-2), and a hospital toilet door handle (HP-10). As a non-enveloped, double-stranded DNA virus, adenovirus exhibits exceptional environmental stability, retaining infectivity for weeks on stainless steel and polypropylene at 21–23 °C and resisting both desiccation and many alcohol-based formulations [60,61]. This stability makes adenovirus one of the pathogens most likely to accumulate on surfaces between cleaning events.

The HP-10 detection (toilet door handle, co-detected with EAEC) is consistent with the “toilet plume” mechanism, whereby virus-laden particles are aerosolized during flushing and deposited on adjacent surfaces, including door handles, taps, and flush buttons [62]. The dual respiratory and fecal–oral transmission capability of adenovirus means that both direct hand contact and toilet plume aerosolization may contribute to surface contamination in hospital restrooms. The mosque surface detection (CP-18) is noteworthy given that mosques in Riyadh are high-frequency, high-density shared-facility environments where multiple individuals make rapid sequential hand-to-surface contact, creating conditions favorable for fomite accumulation. The non-enveloped adenovirus capsid also confers resistance to many alcohol-based hand sanitizers, meaning that hand hygiene compliance alone may be insufficient to interrupt transmission in high-density worship settings without concurrent surface disinfection with non-alcohol agents.

Rhinovirus/Enterovirus (3 pools: HP-6, AP-5, AP-9)

Rhinovirus/Enterovirus was detected in a hospital children’s play area (HP-6), an airport toilet doorknob (AP-5), and an airport food court table (AP-9, co-detected with SARS-CoV-2). As a non-enveloped virus, rhinovirus persists on hard surfaces for hours to days, with greater stability than enveloped viruses under the low-humidity conditions characteristic of air-conditioned indoor environments [63]. The hospital play area detection is consistent with high shedding from pediatric populations, who are the primary reservoir for rhinovirus and frequently contaminate shared toys and surfaces through nasal secretions and hand contact.

The airport detections (AP-5, AP-9) are consistent with traveler-mediated introduction of respiratory pathogens. The co-detection of rhinovirus and SARS-CoV-2 in a single airport pool (AP-9) demonstrates the convergence of multiple respiratory viruses on high-touch surfaces in transit environments, consistent with airports functioning as mixing hubs where geographically distinct respiratory virus lineages are introduced simultaneously by arriving passengers.

Coronavirus 229E (1 pool: AP-7)

Human coronavirus 229E was detected on an airport ATM (AP-7, co-detected with *Cryptosporidium*). The detection of a seasonal coronavirus in an airport environment is consistent with the virus being introduced via international travel. Surface persistence studies show that 229E can survive up to 9 days on aluminum and glass surfaces at 21 °C under laboratory conditions, with markedly faster inactivation above 30 °C [64]. The climate-controlled airport terminal, maintained at approximately 22–24 °C, represents conditions near the laboratory optimum for 229E surface persistence, contextualizing the detection within the Indoor City framework. The co-detection with *Cryptosporidium* indicates convergent contamination from independent sources—respiratory virus introduction via a traveler and fecal–hand transfer of oocysts—on a shared high-touch interface.

*Cryptosporidium* spp. (6 pools: CP-5, CP-20, BPI-3, BPI-5, BPO-5, AP-7)

*Cryptosporidium* was the most frequently detected non-viral pathogen and the only pathogen detected in all three ATM-positive pools (BPI-3, BPI-5, BPO-5), though it was also found in community (CP-5, CP-20) and airport (AP-7) settings. Detection is consistent with fecal–hand–surface transfer via inadequate post-toilet hand hygiene. Oocysts are deposited on keypads by contaminated fingers, and the thick-walled oocyst structure (4–6 µm) confers extreme resistance to desiccation, heat, chlorine, and alcohol-based sanitizers [65]. This structural resilience explains the selective persistence of *Cryptosporidium* on ATM surfaces, where enveloped viruses would degrade within hours under Riyadh’s ambient outdoor temperatures.

The most distinctive site–pathogen pattern in this dataset was the exclusive *Cryptosporidium*-only profile of ATM-positive pools: all three ATM pools (BPI-3, BPI-5, BPO-5) contained *Cryptosporidium* and no other pathogen. Where alcohol-based wipes are used for surface maintenance, they are ineffective against protozoan oocysts, potentially creating a selective survival advantage: routine cleaning may reduce competing pathogens while leaving *Cryptosporidium* intact. This finding has direct infection prevention implications, as discussed in Section 4.3 below. The detection of *Cryptosporidium* in community (CP-5, CP-20) and airport (AP-7) pools is consistent with the ubiquitous fecal–oral route operating wherever hand hygiene is suboptimal after restroom use.

*Vibrio cholerae* (3 pools: CP-13, CP-16, CP-17)

*V. cholerae* was detected exclusively in community settings (Central and West Riyadh parks and cafes), with all three detections occurring in March 2023. This temporal clustering may reflect a shared environmental contamination source or a concurrent contamination event rather than independent sporadic detections. The fecal–oral route is the established transmission pathway; surface detection in food-handling areas is consistent with contamination from hands soiled after restroom use or from handling contaminated food items. *V. cholerae* survives on surfaces for hours to days, depending on moisture and organic matter, with less resistance to desiccation than oocysts. The co-detections with EAEC (CP-13) and SARS-CoV-2 (CP-17) indicate convergent contamination from both fecal–oral and respiratory routes on these community surfaces.

EAEC, EPEC, and *Salmonella* (community and hospital pools)

Enteroaggregative *E. coli* (EAEC; 3 pools: CP-13, CP-20, HP-10), enteropathogenic *E. coli* (EPEC; 1 pool: CP-25), and *Salmonella* spp. (1 pool: CP-9) were detected primarily in community settings, with one hospital detection (EAEC on HP-10, a toilet door handle). These organisms serve as fecal indicator markers, confirming inadequate hand hygiene after restroom use as a prevalent contamination pathway. The hospital toilet door detection (HP-10, co-detected with adenovirus) is particularly consistent with the toilet plume aerosolization mechanism depositing both respiratory and enteric pathogens on restroom surfaces. Community detections implicate food-handling and sanitation gaps in shared public spaces. Appropriately selected and correctly applied surface disinfectants are generally effective against these bacteria, indicating that improved cleaning frequency and hand hygiene infrastructure (soap, water, dispensers) at community sites are the primary control levers.

*Bordetella pertussis* (1 pool: AP-1)

*B. pertussis* was detected in a single airport pool. Environmental surface detection of *B. pertussis* is rarely documented, as the organism has limited environmental persistence and is sensitive to desiccation. The detection likely reflects recent respiratory shedding by a traveler, consistent with the airport’s role as a hub for the introduction of respiratory pathogens from diverse geographic origins. While surface transmission of *B. pertussis* is considered a minor route relative to droplet transmission, the detection underscores that airports serve as convergence points for a broad range of respiratory pathogens beyond the commonly surveilled influenza and coronaviruses.

### 4.3. Site-Specific Infection Prevention and Control Implications

These evidence-based recommendations translate the molecular findings into actionable protocols for specific end-users, including hospital Infection Prevention and Control (IPC) teams, airport facility management, municipal cleaning contractors, and ATM maintenance providers. The stratification of pathogen detections by site type provides a basis for targeted, evidence-informed infection prevention recommendations adapted to the arid indoor-city context of Riyadh (Figure S5).

Hospitals

Hospital detections were dominated by respiratory pathogens (adenovirus, rhinovirus/enterovirus), with one enteric indicator detection (EAEC) on a toilet door handle. Cleaning protocols should employ disinfectants with documented efficacy against both enveloped and non-enveloped viruses, as standard alcohol-based products are suboptimal for non-enveloped adenovirus [66,67]. Cleaning frequency should be increased for waiting area seating and toilet door handles, with particular attention to pediatric areas where rhinovirus shedding is highest. The co-detection of adenovirus and EAEC on a toilet door handle (HP-10) supports the recommendation that hospital restroom surfaces be treated as dual-risk sites requiring disinfection active against both respiratory and enteric organisms.

Airports

Airports exhibited the broadest pathogen diversity per site type, including respiratory viruses (SARS-CoV-2, rhinovirus, CoV-229E), *Cryptosporidium*, and *B. pertussis*. Intensified disinfection protocols may be warranted for toilet doorknobs, food court tables, and ATM keypads during and after peak passenger traffic periods associated with Hajj, Umrah, and Riyadh Season [68]. Environmental surveillance could be integrated into public health screening at international arrival points, and the baseline contamination rates established in this study may inform future exploratory threshold development for triggering enhanced cleaning responses. Touchless alternatives for high-frequency interfaces (payment terminals, check-in kiosks) merit consideration.

ATMs and Banking Surfaces

The finding that all ATM-positive pools contained *Cryptosporidium* only represents the most actionable infection prevention signal in this dataset. If alcohol-based wipes are used for routine ATM maintenance, they are ineffective against protozoan oocysts [69]. Facilities could transition to oocysticidal agents with demonstrated efficacy: hydrogen peroxide-based disinfectants [69], high-concentration sodium hypochlorite [70], or UV-C irradiation [69] for routine ATM cleaning. Installation of hand sanitizer stations adjacent to ATM kiosks may serve as a complementary measure. The acceleration of contactless and NFC payment technology in Saudi Arabia’s Vision 2030 digital transformation agenda offers a structural solution that reduces the reliance on shared keypads entirely.

Community Sites

Community settings exhibited the greatest pathogen diversity (7 of 10 detected pathogens), including enteric pathogens (*V. cholerae*, *Salmonella*, EAEC, EPEC, *Cryptosporidium*) alongside SARS-CoV-2 and adenovirus. Improved sanitation targeting enteric pathogens in public parks and cafes, public education on hand hygiene after using shared surfaces, and municipal coordination on restroom maintenance are the primary interventions. The temporal clustering of *V. cholerae* in March 2023 merits further investigation of water quality at the implicated community sites.

Seasonal Adaptation

A critical operational implication of the Indoor City framework is that cleaning intensification for respiratory viruses should not be limited to winter months. In Riyadh, the finding that 100% of warm-season detections were viral—a pattern consistent with increased indoor crowding during extreme outdoor heat—indicates that summer likely represents a period of elevated respiratory virus contamination risk on indoor surfaces. Infection prevention protocols should therefore align with indoor occupancy cycles (school terms, Ramadan evening economy, summer commercial activity) rather than outdoor meteorological seasons.

### 4.4. Study Limitations

Molecular Detection and Viability

All detections in this study were based on nucleic acid amplification and therefore indicate the presence of pathogen genetic material rather than infectious organisms. PCR detects both intact and degraded particles; viability assays such as cell culture or propidium monoazide (PMA)–PCR provide closer estimates of infectious risk but are more laborious, less sensitive, and inapplicable to non-culturable organisms, including norovirus and *Cryptosporidium* [71]. For enveloped viruses such as SARS-CoV-2, RNA persistence on surfaces substantially exceeds infectivity: viral RNA remains detectable for days after culturable virus is no longer recoverable [72]. The high Ct values observed for most detections (>35 for 7 of 10 detected pathogen categories) suggest that the majority represent non-viable nucleic acid remnants rather than actively infectious material. However, SARS-CoV-2 detections at Ct 33.9–34.8 may indicate more recent contamination events with higher residual pathogen loads. Importantly, Ct interpretation is assay-, matrix-, and target-dependent, and no universal Ct threshold can reliably infer infectivity from environmental surface samples.

Despite this limitation, environmental molecular surveillance—whether applied to surfaces, air, or wastewater—is an internationally recognized tool for public health monitoring and outbreak early warning that relies on nucleic acid detection by design [73]. Detection of pathogen nucleic acid on a surface provides actionable information even in the absence of viability data: it confirms that contamination pathways exist, identifies hygiene failures at specific locations, and provides early signals of pathogen circulation in a community before clinical cases accumulate. For structurally robust organisms—*Cryptosporidium* oocysts, adenovirus capsids—nucleic acid detection correlates more strongly with viability than for fragile enveloped viruses, because the structural integrity that protects nucleic acid also preserves infectivity. Viability assessment using cell culture and PMA-PCR for selected pathogens is identified as a priority for future confirmatory studies. Specifically, PMA-PCR can distinguish intact from membrane-compromised organisms without culture, while cell culture remains the reference standard for culturable viruses; combining both approaches would better quantify infectious risk from environmental surface samples.

Pooling Strategy

The 5-sample pooling strategy improved feasibility and coverage but prevents attribution of positive results to specific individual swabs within a pool. At the expected low background positivity (<5%), the theoretical sensitivity loss from pooling is marginal (approximately 2.3 Ct shift); however, empirical validation of pooling sensitivity was not performed with this specific sample matrix. The pooled design is therefore appropriate for baseline surveillance and hypothesis generation but not for individual-surface risk quantification.

Sample Size and Statistical Power

Of 55 pooled samples, 19 were positive, yielding 10 viral-positive and 9 non-viral-positive pools for the binary logistic regression. The study was not formally powered to detect the observed effect size, and the sample of positive pools (*n* = 19) constrains statistical power for multivariable models and subgroup analyses. The statistically significant result (*p* = 0.032) should therefore be interpreted as hypothesis-generating rather than confirmatory. Statistically non-significant results (e.g., humidity as a predictor) should be interpreted as “insufficient evidence” rather than “evidence of no effect.” Future studies with larger sample sizes across multiple seasons are needed to confirm these preliminary associations.

Meteorological Measurement

Temperature and humidity were measured as outdoor ambient conditions at or near sampling time. The Indoor City framework highlights the structural mismatch between outdoor meteorological variables and the indoor microenvironments where pathogen persistence actually occurs. Future surveillance should incorporate indoor temperature and humidity monitoring as primary predictors rather than relying exclusively on outdoor data.

Environmental Variables and Analytical Scope

While we collected data on broader meteorological variables—including UV index, AQI, wind speed, and pollen—the sample size of 19 positive pools precluded the application of complex multivariable or machine learning models, which would risk severe overfitting and yield unstable estimates. Exploratory analyses of these variables are presented in Figure S3. Future large-scale surveillance efforts should integrate these additional variables with advanced predictive modeling to build on the baseline established here.

Generalizability

The study was conducted in a single city during a defined post-pandemic period (February 2023–May 2024). Context-specific factors—building design, HVAC configurations, cleaning regimes, population density—may limit transferability of findings to other arid megacities, though the theoretical framework may be broadly applicable to Gulf Cooperation Council (GCC) cities with similar climate and infrastructure profiles.

### 4.5. Future Directions: Toward Predictive Environmental Surveillance

The significant associations between temperature and viral detection (OR = 1.728, *p* = 0.032), UV index category and viral detection (Fisher’s exact *p* = 0.036), and the elevated co-detection frequency (31.6% observed vs. 19.8% expected), suggest that surface contamination is governed by multi-factor interactions rather than single-variable drivers. This motivates a shift from univariate approaches toward integrative predictive frameworks. Environmental trend analyses across all sampling periods are presented in Figure S7.

Machine learning algorithms have been increasingly applied to model non-linear relationships between environmental factors and pathogen dynamics. Ensemble methods (gradient boosting, random forest) and Gaussian process regression have demonstrated moderate explanatory power for predicting respiratory disease occurrence from climate and air pollution data [74]. Explainable AI approaches, particularly SHAP (SHapley Additive exPlanations) analysis, can identify which environmental variables contribute most to model predictions, providing interpretable outputs suitable for public health decision-making [75]. Hybrid approaches combining ML with compartmental epidemiological models (e.g., SIR frameworks) show promise for handling time-dependent environmental influences [76].

We propose a stage-by-stage framework, building on the baseline established here. Stage 1 (feature expansion): future studies should incorporate lagged environmental effects (7–21-day windows for temperature, humidity, UV, AQI), composite indices, spatial connectivity metrics, and temporal features (Hajj/Umrah calendars, school terms). Stage 2 (model development): this stage comprises the use of a substantially larger dataset (e.g., hundreds of pools) and penalized regression (LASSO, elastic net) for variable selection, followed by the application of ensemble methods for non-linear interaction capture; the current dataset (N = 55, 19 positive) provides baseline prevalence and effect sizes for power calculation but is insufficient for stable model training. Stage 3 (deployment): this stage involves model outputs translated into operational risk scores compatible with existing municipal cleaning schedules, enabling proactive risk management aligned with Saudi Vision 2030 public health objectives.

Several challenges must be acknowledged. Data quality remains a primary constraint: the current study’s reliance on outdoor ambient conditions rather than indoor microenvironmental measurements introduces measurement error. While correlations between environmental variables and pathogen detection exist, establishing direct causal links remains complex due to multiple mediating behavioral pathways. Interpretability must be prioritized: complex models can function as opaque predictors that limit their utility for operational public health decisions. Future studies should incorporate indoor temperature and humidity monitoring, direct PM_2_._5_/PM_10_ measurements, and foot traffic data as primary predictors.

## 5. Conclusions

This study provides the first city-wide molecular baseline of surface pathogen contamination across diverse public and clinical environments in an arid megacity. The overall positivity rate of 34.5% across 55 pooled samples demonstrates frequent molecular evidence of pathogen contamination on Riyadh’s urban surfaces despite the common assumption that extreme arid heat sharply reduces environmental pathogen persistence. The detection of 10 distinct pathogens—4 respiratory viruses, 1 parasite, and 5 bacterial pathogens—at the species or genus level reveals contamination signatures that are both site-specific and seasonally structured.

The statistically significant association between higher ambient temperature and viral detection among positive pools (OR = 1.728, *p* = 0.032; AUC = 0.756) provides preliminary empirical support consistent with the Indoor City hypothesis: in arid megacities where extreme outdoor heat drives populations into climate-controlled indoor spaces, indoor surfaces serve as recurring sites of respiratory virus contamination even during peak summer. This framework challenges conventional seasonality models and has direct implications for the timing and targeting of infection prevention efforts in Gulf cities.

The following evidence-informed recommendations emerge from the pathogen–site associations identified in this study. Hospitals could employ broad-spectrum disinfectants effective against both enveloped and non-enveloped viruses, with increased cleaning frequency in waiting areas and pediatric zones. Airports may warrant enhanced disinfection during peak passenger periods (Hajj, Umrah, Riyadh Season) with consideration of environmental surveillance as a complementary input to public health screening and adoption of touchless interfaces. ATM disinfection protocols could transition from alcohol-based wipes to oocysticidal agents (hydrogen peroxide, sodium hypochlorite, or UV-C irradiation), complemented by hand sanitizer stations and accelerated adoption of contactless payment technology. Community sites would benefit from improved sanitation targeting enteric pathogens, public hand hygiene education, and coordinated municipal restroom maintenance.

A critical operational implication of the Indoor City framework is that cleaning intensification for respiratory viruses could be aligned with indoor occupancy cycles—school terms, Ramadan evening economy, summer commercial activity—rather than outdoor meteorological seasons. In arid megacities, peak summer indoor crowding likely represents a period of elevated respiratory virus contamination risk on indoor surfaces that conventional winter-focused protocols do not address.

Establishing baseline surface contamination profiles, as demonstrated here, provides the foundation for sentinel environmental surveillance systems capable of detecting deviations from baseline that may signal emerging outbreaks. Integration of environmental monitoring data with clinical surveillance through digital health platforms represents a future priority for enhancing public health preparedness in Saudi Arabia and comparable arid urban environments worldwide.

## Figures and Tables

**Table 1:** Sampling distribution, site coverage, and pooling strategy across Riyadh environmental settings (N = 270).

Sampling Stratum	Targeted Sites & Surfaces	Total Swabs (*n*)	Pools (*n*)	Swabs per Pool	Pool Code Range
Community Regions (North, South, East, West, Central)	Sites: Parks, mosques, supermarkets, restaurants, coffee shops. Surfaces: Play equipment, door handles, cart handles, payment terminals, tables.	100	25	4	CP-1 to CP-25
Healthcare Hub (King Fahad Medical City)	Sites: Waiting areas, reception, clinics, restrooms. Surfaces: Armchairs, counters, door handles, sinks, flush buttons.	70	10	7	HP-1 to HP-10
Transportation Hub (King Khalid Int. Airport)	Sites: Check-in, food court, restrooms. Surfaces: Counters, luggage trolleys, escalator handrails, tables.	50	10	5	AP-1 to AP-10
Financial Services (Indoor)	Sites: Bank branches. Surfaces: ATM keypads, screens, card slots (indoor climate).	25	5	5	BPI-1 to BPI-5
Financial Services (Outdoor)	Sites: Drive-through or street-side kiosks. Surfaces: ATM interfaces (exposed to outdoor elements).	25	5	5	BPO-1 to BPO-5
Total		270	55		

Note: The study area encompassed five geographic zones in Riyadh plus two major institutional hubs. Abbreviations: CP, Community Pool; HP, Hospital Pool; AP, Airport Pool; BPI, Bank Pool Indoor; BPO, Bank Pool Outdoor. Samples were pooled prior to DNA/RNA extraction to optimize molecular surveillance efficiency.

**Table 2:** Distribution of pooled environmental samples and overall pathogen prevalence by study setting (N = 55).

Pool Group	Setting Category	Targeted Sites	Pools (*n*)	Swabs per Pool	Total Swabs	Positive Pools *n* (%)
CP	Community Hubs	Restaurants, parks, mosques, markets	25	4	100	9 (36.0%)
HP	Healthcare Hub	Hospital (KFMC)	10	7	70	3 (30.0%)
AP	Transportation Hub	Airport (KKIA)	10	5	50	4 (40.0%)
BPI	Financial (Indoor)	Bank ATMs (Indoor)	5	5	25	2 (40.0%)
BPO	Financial (Outdoor)	Bank ATMs (Outdoor)	5	5	25	1 (20.0%)
Total	All Settings		55	4–7	270	19 (34.5%)

Note: Individual surface swabs (N = 270) were aggregated into 55 pools prior to molecular testing.

**Table 3:** Pathogen-specific detection profile, plausible circulation pathways, and recommended control measures in arid urban conditions.

Pathogen (Classification)	Pools Detected (*n*)	Pool IDs	Primary Site(s) and Surface(s)	Ct Range	Plausible Circulation Pathway	Persistence in Arid Conditions	Recommended Control Measures
SARS-CoV-2 (*Enveloped RNA virus*)	4	CP-8, CP-17, CP-20, AP-9	Community (supermarket carts, park benches, coffee shop tables); Airport (food court table)	33.9–34.8	Respiratory droplet deposition and direct hand contact from symptomatic/pre-symptomatic individuals. Indoor HVAC systems may redistribute aerosolized virus to distal surfaces. Co-detection with *V. cholerae* (CP-17) and *Cryptosporidium* + EAEC (CP-20) is consistent with convergent multi-source contamination at high-traffic community surfaces.	Enveloped; rapid inactivation outdoors at >40 °C (half-life < 2 h at 27 °C). Persists hours to days on non-porous surfaces at indoor AC temperatures (20–24 °C). RNA outlasts infectivity.	Standard virucidal disinfectants (alcohol, QUATs, sodium hypochlorite). Increase cleaning frequency of indoor high-touch surfaces during peak summer months (indoor crowding). Improve ventilation in enclosed public spaces.
Adenovirus (*Non-enveloped DNA virus*)	3	CP-18, HP-2, HP-10	Community (mosque door handle); Hospital (waiting area seat, toilet door handle)	36.2–38.3	Dual respiratory and fecal–oral transmission. “Toilet plume” aerosolization during flushing deposits virus on adjacent surfaces (HP-10 toilet door). Fomite contact in high-density mosque environments (CP-18). Co-detected with EAEC in HP-10.	Non-enveloped dsDNA capsid; exceptional environmental stability. Persists weeks on stainless steel and plastic at 21–23 °C. Resistant to desiccation and many alcohol formulations.	Non-alcohol disinfectants required (sodium hypochlorite, oxidative agents). Enhanced cleaning of toilet doors, waiting area seating, and communal worship surfaces. Targeted monitoring of pediatric hospital areas.
Rhinovirus/ *Enterovirus*	3	HP-6, AP-5, AP-9	Hospital (children’s play area); Airport (toilet doorknob, food court table)	34.2–37.9	Respiratory secretion deposition via hand contact. High shedding from pediatric populations in play areas (HP-6). Airport detections consistent with traveler-mediated introduction. Co-detected with SARS-CoV-2 in AP-9.	Non-enveloped; persists on hard surfaces for hours to days. More robust than enveloped viruses under low-humidity conditions.	Routine surface disinfection with broad-spectrum virucidal agents. Prioritize children’s play areas and airport high-touch surfaces (doorknobs, food court tables). Hand hygiene education.
Coronavirus 229E (*Enveloped RNA virus*)	1	AP-7	Airport (ATM)	37.8	Seasonal coronavirus likely introduced via international travel. Detected in climate-controlled airport terminal where indoor temperatures (~22–24 °C) favor 229E persistence. Co-detected with *Cryptosporidium*, indicating convergent contamination from independent sources.	Enveloped; survives up to 9 days on aluminum/glass at 21 °C in lab conditions. Rapid inactivation above 30 °C. AC environments maintain near-optimal conditions.	Standard virucidal disinfection of airport ATMs and touchscreens. Enhanced protocols during Hajj/Umrah periods. Consider touchless payment alternatives.
*CryptosporidiumCryptosporidium* spp. (*Protozoan parasite—oocyst*)	6	CP-5, CP-20, BPI-3, BPI-5, BPO-5, AP-7	ATM indoor (bank branch); ATM outdoor (street kiosk); Community; Airport (ATM)	34.6–37.9	Fecal–hand–surface transfer via inadequate post-toilet hand hygiene. Oocysts deposited on keypads by contaminated fingers. *Cryptosporidium* was the only pathogen detected in ATM pools (all 3 ATM-positive pools: BPI-3, BPI-5, BPO-5), but it was also detected in community (CP-5, CP-20) and airport (AP-7) pools, indicating broad environmental dissemination.	Thick-walled oocyst (4–6 µm); extreme resistance to desiccation, heat, chlorine, and alcohol. Survives months in environmental matrices. Most persistent organism in the dataset.	CRITICAL: Alcohol-based wipes are INEFFECTIVE. Require oocysticidal agents: hydrogen peroxide-based disinfectants, high-concentration sodium hypochlorite, or UV-C irradiation. Install hand sanitizer stations at ATMs. Accelerate contactless/NFC payment technology.
*Vibrio cholerae* (*Gram-negative bacterium* )	3	CP-13, CP-16, CP-17	Community (Central and West Riyadh parks, cafes)	36.8–37.9	Fecal–oral route; typically waterborne but surface detection reported in food-handling areas. Temporally clustered (all March 2023), suggesting shared environmental source or concurrent contamination event rather than independent sporadic detections. Co-detected with EAEC (CP-13) and SARS-CoV-2 (CP-17).	Survives on surfaces hours to days depending on moisture and organic matter. Less resistant to desiccation than oocysts but can persist in moist/organic matrices.	Improve food-handling hygiene and sanitation in public parks/cafes. Investigate water quality at implicated community sites. Standard disinfection protocols effective.
*EAEC* (*Enteroaggregative E. coli*)	3	CP-13, CP-20, HP-10	Community; Hospital (toilet door handle)	37.5–38.2	Fecal–oral transmission via contaminated hands. Fecal indicator organism confirming inadequate hand hygiene after restroom use. Co-detected with *V. cholerae* (CP-13), SARS-CoV-2 + *Cryptosporidium* (CP-20), and Adenovirus (HP-10).	Moderate environmental persistence on non-porous surfaces. Susceptible to standard disinfectants.	Enhanced restroom sanitation. Hand hygiene infrastructure (soap, water, dispensers). Standard disinfection of toilet door handles and communal surfaces.
*EPEC* (*Enteropathogenic E. coli*)	1	CP-25	Community	35.7	Fecal–oral route. Indicator of fecal contamination on shared community surfaces.	Similar to EAEC. Moderate persistence on hard surfaces.	As for EAEC above.
*Salmonella* spp. (*Gram-negative bacterium*)	1	CP-9	Community	37.2	Foodborne/fecal–oral route. Detection in community settings implicates food-handling gaps or cross-contamination in shared dining areas.	Persists days to weeks on dry surfaces. Biofilm formation can enhance survival.	Improve food safety practices in community food service areas. Standard surface disinfection. Municipal coordination on food vendor hygiene.
*Bordetella pertussis* (*Gram-negative bacterium*)	1	AP-1	Airport	36.5	Respiratory droplet deposition. Rarely documented on environmental surfaces. Airport detection suggests respiratory shedding by a traveler. Only bacterial detection in the airport setting.	Limited environmental persistence; sensitive to desiccation. Detection likely reflects recent contamination.	Routine surface disinfection. Vaccination remains primary prevention. Detection highlights airport role as respiratory pathogen introduction hub.

Note: Ct, cycle threshold (lower values = higher pathogen load). All detections via QIAstat-Dx Respiratory SARS-CoV-2 Panel and/or Gastrointestinal Panel 2. Pooled samples (4–5 swabs per pool). Row shading: blue = viral, purple = parasitic, yellow = bacterial. All Ct values and pool IDs verified against QIAstat-Dx primary data. Abbreviations: AC, air conditioning; ATM, automated teller machine; dsDNA, double-stranded DNA; EAEC, enteroaggregative *E. coli*; EPEC, enteropathogenic *E. coli*; HVAC, heating, ventilation, and air conditioning; NFC, near-field communication; QUATs, quaternary ammonium compounds; UV-C, ultraviolet-C.

**Table 4:** Surface contamination risk ranking by positivity rate, pathogen diversity, and co-detection frequency.

Rank	Surface Type	Pos/Total	Rate	Species Detected	Co-Detection	Pathogens Detected
1	Restroom surfaces (door handles, taps, flush buttons)	12/27	44.4%	10	5/12 (42%)	SARS-CoV-2, Adenovirus, Rhinovirus/Enterovirus, CoV-229E, *V. cholerae*, EAEC, EPEC, *Salmonella*, *Cryptosporidium*, *B. pertussis*
2	ATM touchscreen (indoor)	2/5	40.0%	1	0/2 (0%)	*Cryptosporidium* (exclusive)
3	Waiting area seating/door handles (hospital)	3/10	30.0%	3	1/3 (33%)	Adenovirus, Rhinovirus/Enterovirus, EAEC
4	ATM touchscreen (outdoor)	1/5	20.0%	1	0/1 (0%)	*Cryptosporidium* (exclusive)

**Table 5:** Recommended disinfection agents by surface type based on pathogen risk profile.

Surface	Primary Risk	Recommended Agent	Rationale
Restrooms	Multi-pathogen (viral + bacterial + parasitic)	Broad-spectrum: H_2_O_2_ or NaOCl (≥1000 ppm)	Only agents effective against both enveloped viruses AND protozoan oocysts
ATM touchscreens	*Cryptosporidium* (exclusive)	Oocysticidal: H_2_O_2_ or UV-C irradiation	Alcohol-based wipes ineffective against oocysts
Hospital waiting areas/doors	Adenovirus + EAEC	Quaternary ammonium + enhanced frequency	Non-enveloped virus persistence requires contact time compliance

**Table 6:** Descriptive and inferential statistics for meteorological variables across result groups.

Variable	Statistic	Negative (*n* = 36)	Non-Viral Positive (*n* = 9)	Viral Positive (*n* = 10)	Test Statistic (H)	*p*-Value
Temperature (°C)	Mean ± SD	29.9 ± 9.4	24.7 ± 9.9	33.6 ± 7.4	3.74	0.154
	Median (IQR)	32.0 (7.4)	31.0 (18.8)	33.2 (13.1)		
Humidity (%)	Mean ± SD	23.9 ± 17.4	27.4 ± 16.6	18.7 ± 10.9	1.14	0.566
	Median (IQR)	17.6 (21.0)	17.6 (29.2)	17.4 (20.6)		

Note: H represents the Kruskal–Wallis test statistic. For statistical comparison, the 4 co-infected pools were assigned to the Viral group. The total descriptive prevalence of non-viral pathogens is 13 pools.

**Table 7:** Binary Logistic Regression Predicting Viral vs. Non-Viral Pathogen Presence from Meteorological Factors.

Variable	OR	95% CI for OR	Wald χ^2^	*p*-Value
Temperature (°C)	1.728	[1.050, 2.845]	4.624	0.032
Humidity (%)	0.008	[0.000, 12.808]	3.602	0.200

The Odds Ratio for humidity is calculated based on a proportion scale (0.0–1.0). An OR of 0.008 reflects the theoretical effect of a 0% to 100% shift in humidity.

## Data Availability

The original contributions presented in this study are included in the article/Supplementary Materials. Further inquiries can be directed to the corresponding author.

## Supplementary Materials

The following supporting information can be downloaded at: https://www.mdpi.com/article/10.3390/microorganisms14030626/s1. Figure S1: Pathogen detection by functional surface zone; Figure S2: Air quality index and pathogen detection; Figure S3: Environmental conditions and pathogen detection: pollen, AQI, and multivariate analysis; Figure S4: Air quality index versus pathogen load among positive pools; Figure S5: Pathogen diversity and co-detection patterns across regions; Figure S6: UV index and pathogen detection; Figure S7: Environmental trend analysis across sampling periods; Table S1: Pool-level metadata.

## Abbreviations

The following abbreviations are used in this manuscript

AP	King Khalid International Airport Pool Samples
ATM	Automated Teller Machine
BPI	Bank Pool Indoor Samples
BPO	Bank Pool Outdoor
CP	Community Pool Samples
EAEC	Enteroaggregative *E. coli*
EPEC	Enteropathogenic *E. coli*
GCC	Gulf Cooperation Council
HP	Hospital Pool Samples
KFMC	King Fahad Medical City
KKIA	King Khalid International Airport
PCR	Polymerase Chain Reaction
PPE	Personal Protective Equipment
RT-PCR	Reverse Transcription Polymerase Chain Reaction
SARS-CoV-2	Severe Acute Respiratory Syndrome Coronavirus 2
VTM	Viral Transport Medium
