# Supplementary material for: Pathogens on High-Touch Surfaces in an Arid Megacity: A Longitudinal Molecular Surveillance Study
Source: Microorganisms. 2026 Mar 10;14(3):626. doi: 10.3390/microorganisms14030626 (PMC13028951; doi:10.3390/microorganisms14030626)
Supplement: Supplementary file 1 [file microorganisms-14-00626-s001.zip › Supplementary_Figures_S1toS7_FINAL (2).pdf]

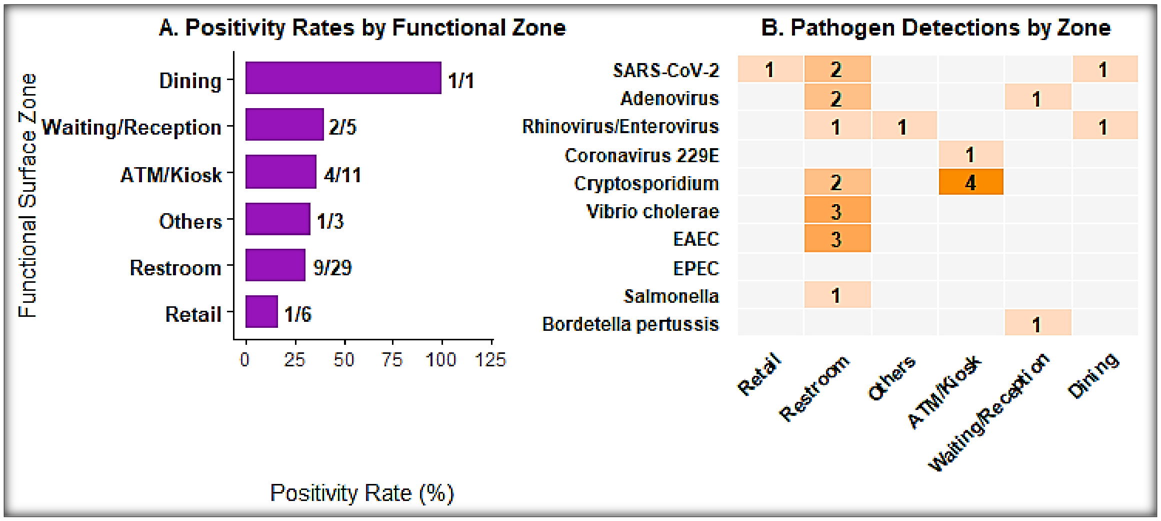

Figure S1. Pathogen detection by functional surface zone. Two-panel figure showing (A) positivity rates by functional zone (horizontal bar chart) and (B) a cross-tabulation heatmap of functional zone × pathogen. Functional zones were derived from the Sample Source descriptions in the master dataset. Zones include Restroom, ATM/Kiosk, Retail, Dining, Door/Handle, Recreation, Waiting/Reception, and others. Counts shown as (positive/total). N = 55 pools; 19 positive.

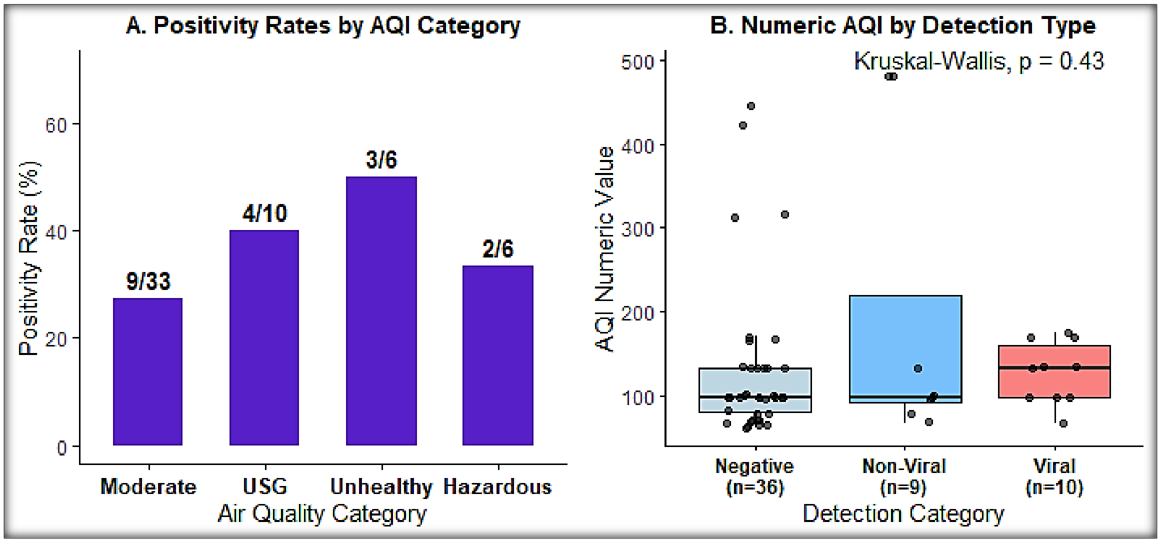

Figure S2. Air quality index and pathogen detection. Two-panel figure. Panel A: positivity rates by AQI category (Moderate, Unhealthy for Sensitive Groups [USG], Unhealthy, Hazardous). Panel B: box-and-whisker plots of AQI numeric values by detection type (Negative, Non-Viral Positive, Viral Positive). AQI data were available for all 55 pools. N = 55 pools; 19 positive.

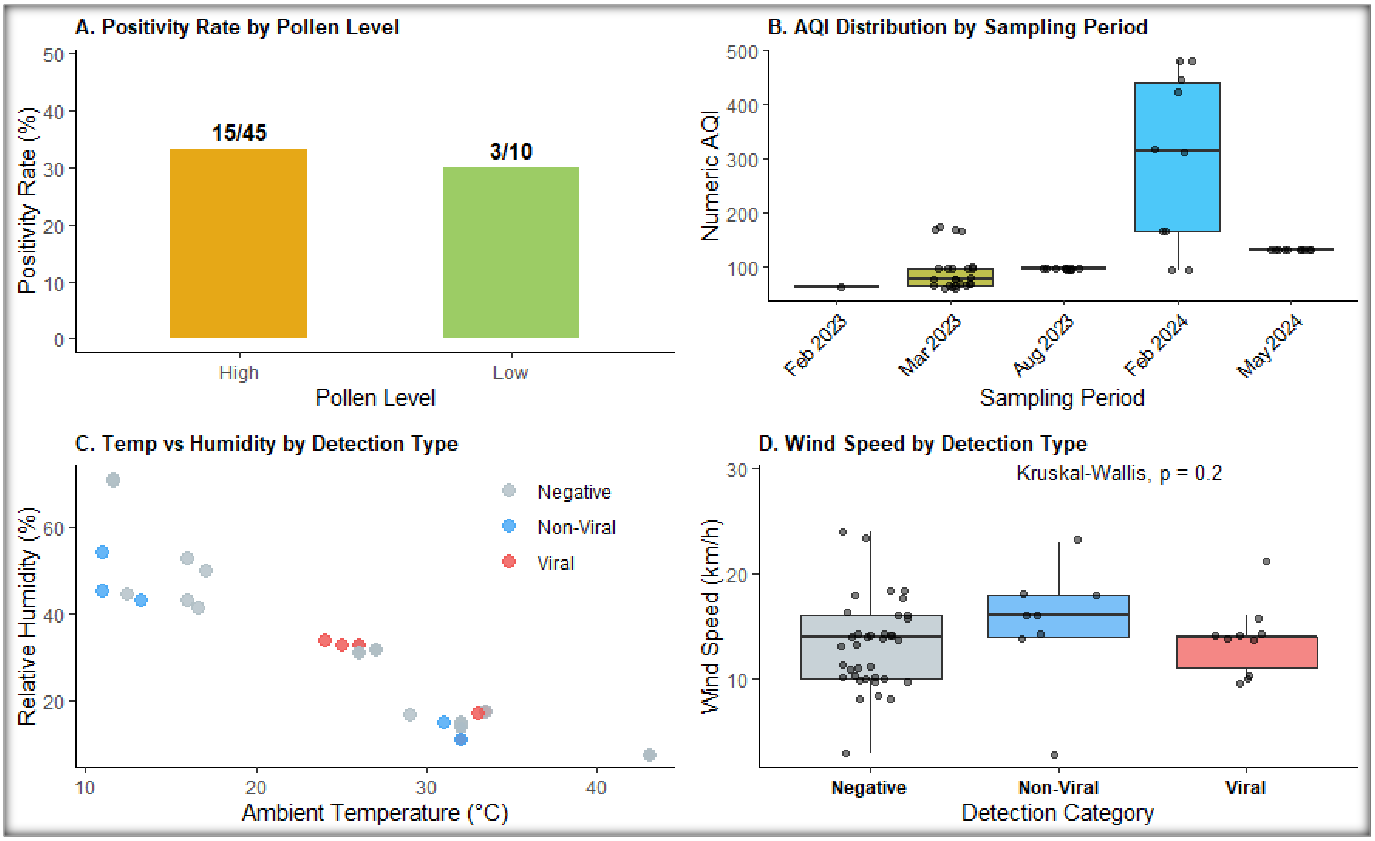

Figure S3. Environmental conditions and pathogen detection: pollen, AQI, and multivariate analysis. Four-panel figure. (A) Positivity rate by pollen level (Low vs. High). (B) AQI distribution by sampling period. (C) Scatter plot of temperature versus humidity colored by detection type. (D) Wind speed boxplots by detection type. N = 55 pools; 19 positive; environmental data available for all pools.

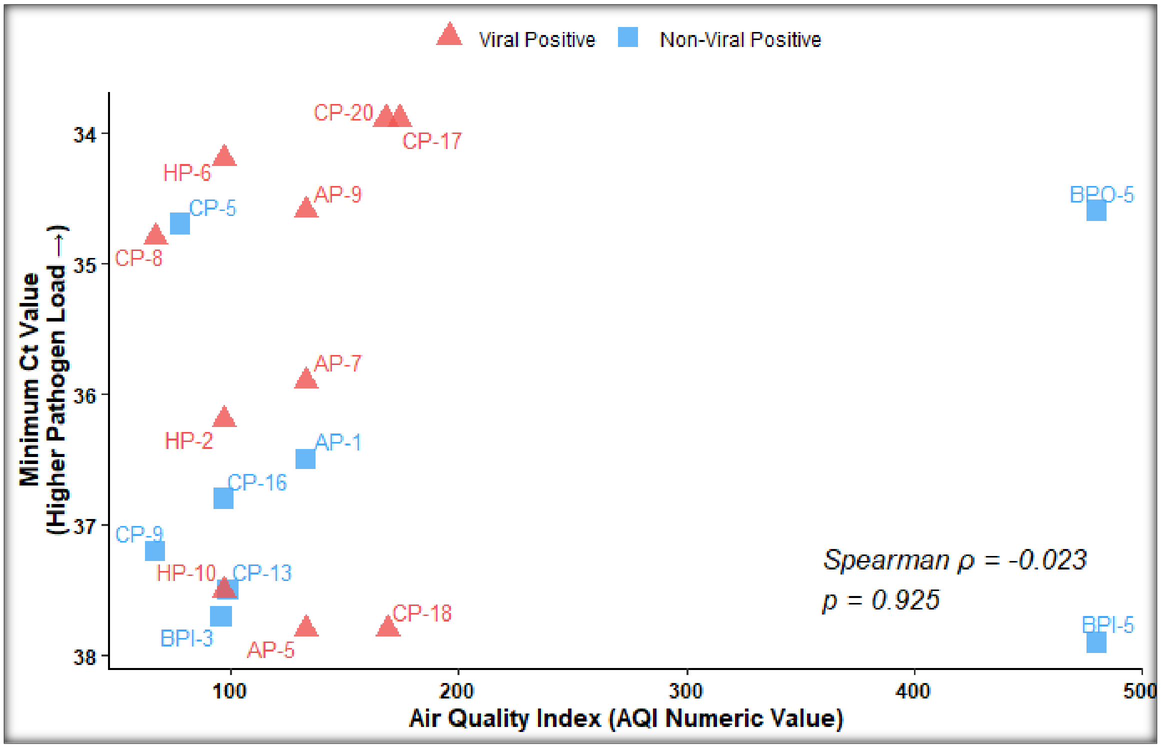

Figure S4. Air quality index versus pathogen load among positive pools. Scatter plot of AQI versus minimum Ct value (inverted y-axis; lower Ct = higher pathogen load) for 19 positive pools. Ct values obtained from QIAstat-Dx panel outputs (Supplementary Table S3). Points colored and shaped by pathogen category: Viral Positive (triangles, n = 10) and Non-Viral Positive (squares, n = 9). Pool IDs annotated for traceability. Spearman $\rho = -0.023$, $p = 0.925$, indicating no significant linear association between AQI and pathogen load.

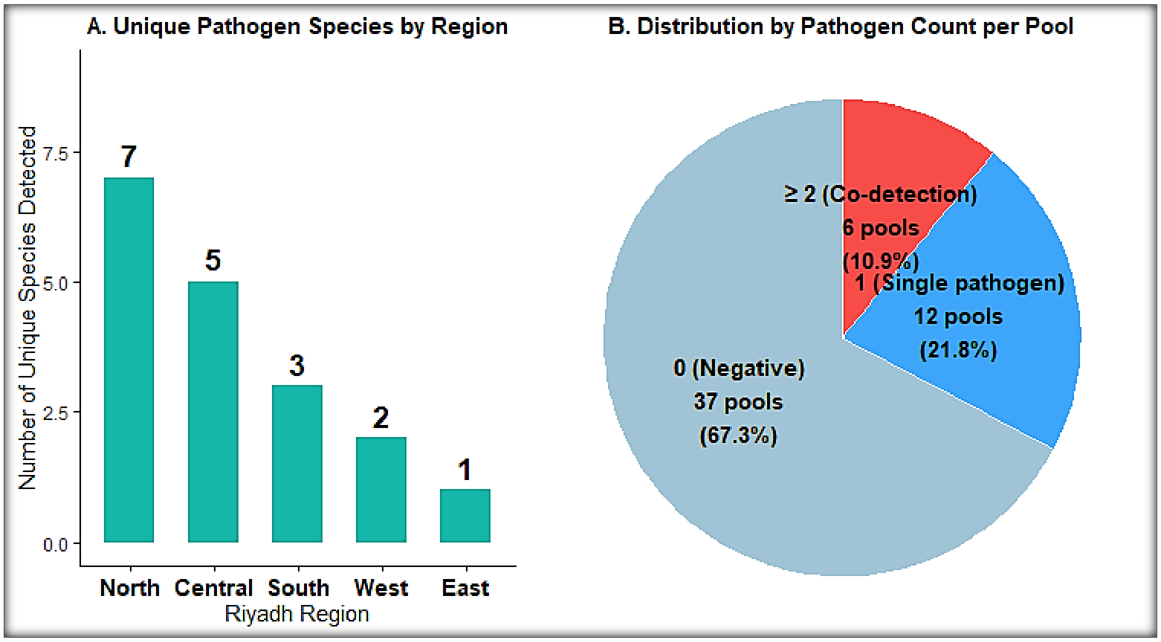

Figure S5. Pathogen diversity and co-detection patterns across regions. Two-panel figure. Panel A: number of unique pathogen species detected per Riyadh region. Panel B: pie chart showing the distribution of pools by pathogen count per pool (0 = negative, 1 = single pathogen, ≥2 = co-detection). N = 55 pools; 19 positive.

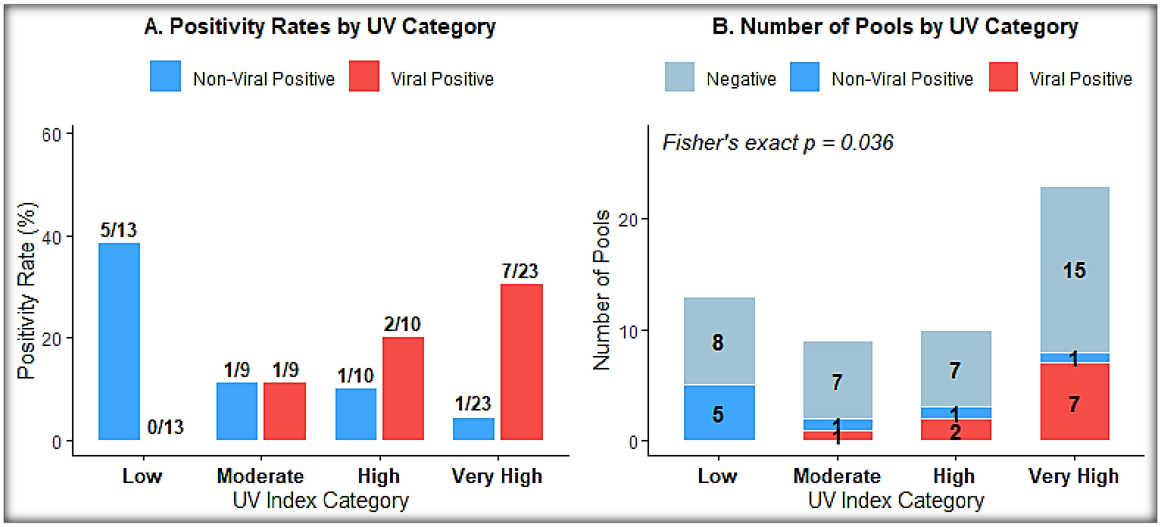

Figure S6. UV index and pathogen detection. Two-panel figure examining the association between UV index category and detection type. Panel A: grouped bars of Viral Positive and Non-Viral Positive rates by UV category (Low, Moderate, High, Very High). Panel B: stacked bars showing the number of Negative, Viral Positive, and Non-Viral Positive pools per UV category. At Low UV, all five detections (100%) were non-viral; viral detections concentrated at High and Very High UV (Fisher exact p = 0.036; see Section 3.6.4). Viral Positive includes mixed viral + non-viral pools. N = 55 pools; 19 positive.

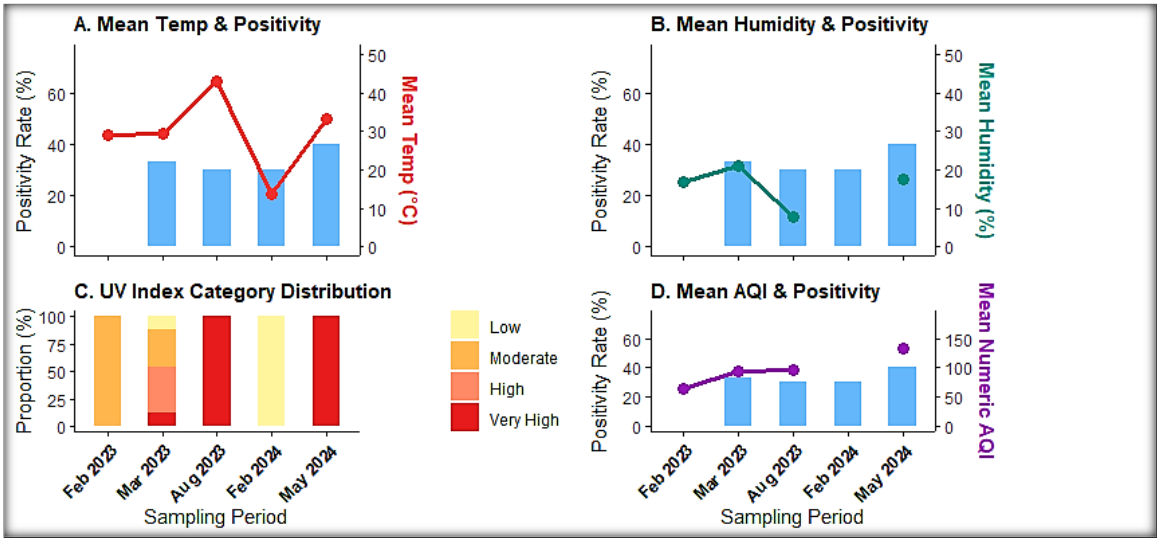

Figure S7. Environmental trend analysis across sampling periods. Four-panel figure showing longitudinal relationships between environmental variables and pathogen detection. (A) Mean temperature (°C) and positivity rate by period. (B) Mean relative humidity (%) and positivity rate by period. (C) UV index category distribution by sampling period. (D) Mean AQI and positivity rate by period. Blue bars = positivity rate; colored lines = environmental variables. Environmental data available for all 55 pools.
