# Supplementary material for: Pathogens on High-Touch Surfaces in an Arid Megacity: A Longitudinal Molecular Surveillance Study
Source: Microorganisms. 2026 Mar 10;14(3):626. doi: 10.3390/microorganisms14030626 (PMC13028951; doi:10.3390/microorganisms14030626)
Supplement: Supplementary file 1 [file microorganisms-14-00626-s001.zip › Supplementary Table S1.pdf]

Table S1. Complete Sampling Metadata — Pool-Level Summary

Summary of 270 environmental swabs collected across seven Riyadh zones (February 2023 – May 2024), aggregated into 55 pools. Each row represents one pool showing constituent swab IDs, venue type, surfaces sampled, meteorological conditions at collection, and QIAstat-Dx screening outcome. Positive pools are shaded.

Total: 270 swabs → 55 pools → 19 positive (34.5%) | ■ **Shaded rows = positive detection**

Community (CP) — 25 pools (100 swabs), 9 positive

Pool ID	n	Constituent Samples	Date	Venue	Surfaces Sampled	Region	°C	RH	Season	QIAstat-Dx Result
CP-1	4	C1, C2, C3, C4	25 Feb 2023	Restaurant	Toilet Sprayer, Toilet Flush, Toilet Door Handle, Toilet Seat	North	29	17%	Winter	Negative
CP-2	4	C5, C6, C7, C8	05 Mar 2023	Supermarket	Shopping Cart, Supermarket Payment Machine, Coke Fridge, Supermarket Weighing Scale	North	32	15%	Winter/Spring	Negative
CP-3	4	C9, C10, C11, C12	05 Mar 2023	Supermarket	Shopping Cart, Supermarket Weighing Scale, Coke Fridge, Supermarket Payment Machine	Central	32	15%	Winter/Spring	Negative
CP-4	4	C13, C14, C15, C16	06 Mar 2023	Park	Toilet Door Handle, Toilet Seat, Toilet Flush, Toilet Sprayer	North	32	14%	Winter/Spring	Negative
**CP-5**	4	C17, C18, C19, C20	06 Mar 2023	Restaurant	Toilet Sprayer, Toilet Seat, Toilet Door Handle, Toilet Flush	East	32	14%	Winter/Spring	**Cryptosporidium**
CP-6	4	C21, C22, C23, C24	06 Mar 2023	Supermarket	Shopping Cart, Supermarket Weighing Scale, Supermarket Payment Machine, Coke Fridge	East	32	14%	Winter/Spring	Negative
CP-7	4	C25, C26, C27, C28	06 Mar 2023	Mosque	Toilet Door Handle, Toilet Sprayer, Toilet Flush, Toilet Seat	East	32	14%	Winter/Spring	Negative
**CP-8**	4	C29, C30, C31, C32	07 Mar 2023	Supermarket	Supermarket Weighing Scale, Coke Fridge, Supermarket Payment Machine, Shopping Cart	South	32	11%	Winter/Spring	**SARS-CoV-2**
**CP-9**	4	C33, C34, C35, C36	07 Mar 2023	Restaurant	Toilet Sprayer, Toilet Door Handle, Toilet Flush, Toilet Seat	South	32	11%	Winter/Spring	**Salmonella**
CP-10	4	C37, C38, C39, C40	07 Mar 2023	Park	Toilet Flush, Toilet Seat, Toilet Door Handle, Toilet Sprayer	South	32	11%	Winter/Spring	Negative
CP-11	4	C41, C42, C43, C44	07 Mar 2023	Mosque	Toilet Seat, Toilet Door Handle, Toilet Flush, Toilet Sprayer	South	32	11%	Winter/Spring	Negative
CP-12	4	C45, C46, C47, C48	07 Mar 2023	Coffee Shop	Toilet Door Handle, Toilet Sprayer, Toilet Flush, Toilet Seat	South	32	11%	Winter/Spring	Negative
**CP-13**	4	C49, C50, C51, C52	08 Mar 2023	Mosque	Toilet Sprayer, Toilet Door Handle, Toilet Seat, Toilet Flush	West	32	14%	Winter/Spring	**Vibrio cholerae; Enteroaggregative E. coli (EAEC)**
CP-14	4	C53, C54, C55, C56	18 Mar 2023	Supermarket	Supermarket Weighing Scale, Coke Fridge, Supermarket Payment Machine, Shopping Cart	West	27	32%	Winter/Spring	Negative
CP-15	4	C57, C58, C59, C60	08 Mar 2023	Restaurant	Toilet Door Handle, Toilet Flush, Toilet Sprayer, Toilet Seat	West	32	15%	Winter/Spring	Negative
**CP-16**	4	C61, C62, C63, C64	08 Mar 2023	Park	Toilet Door Handle, Toilet Flush, Toilet Seat, Toilet Sprayer	West	31	15%	Winter/Spring	**Vibrio cholerae**
**CP-17**	4	C65, C66, C67, C68	11 Mar 2023	Park	Toilet Door Handle, Toilet Seat, Toilet Flush, Toilet Sprayer	Central	24	34%	Winter/Spring	**SARS-CoV-2; Vibrio cholerae**
**CP-18**	4	C69, C70, C71, C72	11 Mar 2023	Mosque	Toilet Door Handle, Toilet Seat, Toilet Flush, Toilet Sprayer	Central	25	33%	Winter/Spring	**Adenovirus**
CP-19	4	C73, C74, C75, C76	11 Mar 2023	Restaurant	Toilet Door Handle, Toilet Sprayer, Toilet Flush, Toilet Seat	Central	26	33%	Winter/Spring	Negative
**CP-20**	4	C77, C78, C79, C80	11 Mar 2023	Coffee Shop	Toilet Door Handle, Toilet Flush, Toilet Seat, Toilet Sprayer	Central	26	33%	Winter/Spring	**SARS-CoV-2; Enteroaggregative E. coli (EAEC); Cryptosporidium**
CP-21	4	C81, C82, C83, C84	17 Mar 2023	Park	Toilet Door Handle, Toilet Flush, Toilet Sprayer, Toilet Seat	East	26	31%	Winter/Spring	Negative

CP-22	4	C85, C86, C87, C88	17 Mar 2023	Coffee Shop	Toilet Door Handle, Toilet Sprayer, Toilet Flush, Toilet Seat	East	26	31%	Winter/Spring	Negative
CP-23	4	C89, C90, C91, C92	17 Mar 2023	Coffee Shop	Toilet Door Handle, Toilet Sprayer, Toilet Flush, Toilet Seat	North	26	31%	Winter/Spring	Negative
CP-24	4	C93, C94, C95, C96	18 Mar 2023	Coffee Shop	Toilet Door Handle, Toilet Sprayer, Toilet Seat, Toilet Flush	West	27	32%	Winter/Spring	Negative
**CP-25**	4	C97, C98, C99, C100	18 Mar 2023	Mosque	Toilet Seat, Toilet Door Handle, Toilet Flush, Toilet Sprayer	North	27	32%	Winter/Spring	**Enteropathogenic E. coli (EPEC)**

Hospital (HP) — 10 pools (70 swabs), 3 positive

Pool ID	n	Constituent Samples	Date	Venue	Surfaces Sampled	Region	°C	RH	Season	QIAstat-Dx Result
HP-1	7	H1, H12, H21, H31, H41, H51, H61	16 Aug 2023	Hospital	Toilet / Sink	North	44	7%	Summer	Negative
**HP-2**	7	H2, H11, H30, H35, H49, H59, H69	16 Aug 2023	Hospital	Waiting Area / Arm Chair	North	44	7%	Summer	**Adenovirus**
HP-3	7	H3, H18, H25, H39, H47, H57, H67	16 Aug 2023	Hospital	Front Desk / Surface	North	44	7%	Summer	Negative
HP-4	7	H4, H16, H27, H37, H45, H55, H65	16 Aug 2023	Hospital	Clinic / Sink	North	44	7%	Summer	Negative
HP-5	7	H5, H17, H28, H38, H46, H56, H66	16 Aug 2023	Hospital	Clinic / Keyboard & Mouse	North	44	7%	Summer	Negative
**HP-6**	7	H6, H20, H29, H36, H50, H60, H70	16 Aug 2023	Hospital	Kids Toys	North	44	7%	Summer	**Rhinovirus/Enterovirus**
HP-8	7	H7, H19, H26, H40, H48, H58, H68	16 Aug 2023	Hospital	Front Desk / Keyboard & Mouse	North	44	7%	Summer	Negative
HP-7	7	H8, H14, H24, H34, H42, H52, H62	16 Aug 2023	Hospital	Toilet / Sprayer	North	44	7%	Summer	Negative
HP-9	7	H9, H15, H23, H33, H43, H53, H63	16 Aug 2023	Hospital	Toilet / Seat	North	44	7%	Summer	Negative
**HP-10**	7	H10, H13, H22, H32, H44, H54, H64	16 Aug 2023	Hospital	Toilet / Door Knob	North	44	7%	Summer	**Adenovirus; Enteroaggregative E. coli (EAEC)**

ATM — Indoor (BPI) — 5 pools (25 swabs), 2 positive

Pool ID	n	Constituent Samples	Date	Venue	Surfaces Sampled	Region	°C	RH	Season	QIAstat-Dx Result
BPI-1	5	B1, B2, B3, B5, B7	25 Feb 2024	ATM / Indoor	ATM / Indoor	North	16	52%	Winter	Negative
BPI-2	5	B12, B13, B15, B17, B19	26 Feb 2024	ATM / Indoor	ATM / Indoor	East	11	73%	Winter	Negative
**BPI-3**	5	B22, B25, B26, B28, B30	27 Feb 2024	ATM / Indoor	ATM / Indoor	Central	12	46%	Winter	**Cryptosporidium**
BPI-4	5	B31, B32, B33, B34, B40	28 Feb 2024	ATM / Indoor	ATM / Indoor	West	15	44%	Winter	Negative
**BPI-5**	5	B41, B42, B43, B44, B48	29 Feb 2024	ATM / Indoor	ATM / Indoor	South	11	58%	Winter	**Cryptosporidium**

ATM — Outdoor (BPO) — 5 pools (25 swabs), 1 positive

Pool ID	n	Constituent Samples	Date	Venue	Surfaces Sampled	Region	°C	RH	Season	QIAstat-Dx Result
BPO-1	5	B4, B6, B8, B9, B10	25 Feb 2024	ATM / Outdoor	ATM / Outdoor	North	16	52%	Winter	Negative
BPO-2	5	B11, B14, B16, B18, B20	26 Feb 2024	ATM / Outdoor	ATM / Outdoor	East	11	73%	Winter	Negative
BPO-3	5	B21, B23, B24, B27, B29	27 Feb 2024	ATM / Outdoor	ATM / Outdoor	Central	12	46%	Winter	Negative

BPO-4	5	B35, B36, B37, B38, B39	28 Feb 2024	ATM / Outdoor	ATM / Outdoor	West	16	44%	Winter	Negative
**BPO-5**	5	B45, B46, B47, B49, B50	29 Feb 2024	ATM / Outdoor	ATM / Outdoor	South	11	49%	Winter	**Cryptosporidium**

Airport (AP) — 10 pools (50 swabs), 4 positive

Pool ID	n	Constituent Samples	Date	Venue	Surfaces Sampled	Region	°C	RH	Season	QIAstat-Dx Result
**AP-1**	5	A1, A11, A22, A32, A41	05 May 2024	KKIA	Check-in Counter	North	31	25%	Spring	**Bordetella pertussis**
AP-2	5	A2, A12, A23, A33, A42	05 May 2024	KKIA	Toilet Sprayer	North	31	25%	Spring	Negative
AP-3	5	A3, A13, A24, A34, A43	05 May 2024	KKIA	Toilet Sink	North	31	25%	Spring	Negative
AP-4	5	A4, A14, A25, A35, A44	05 May 2024	KKIA	Toilet Seat	North	31	25%	Spring	Negative
**AP-5**	5	A5, A15, A26, A36, A45	05 May 2024	KKIA	Toilet Doorknob	North	31	25%	Spring	**Rhinovirus/Enterovirus**
AP-6	5	A6, A16, A27, A37, A46	05 May 2024	KKIA	Luggage Trolley	North	31	25%	Spring	Negative
**AP-7**	5	A7, A17, A28, A38, A47	05 May 2024	KKIA	ATM	North	31	25%	Spring	**Coronavirus 229E; Cryptosporidium**
AP-8	5	A8, A18, A29, A39, A48	05 May 2024	KKIA	Information Desk	North	31	25%	Spring	Negative
**AP-9**	5	A9, A19, A30, A49, A51	05 May 2024	KKIA	Food Court Tables and Chair	North	31	25%	Spring	**Rhinovirus/Enterovirus; SARS-CoV-2**
AP-10	5	A10, A20, A31, A40, A50	05 May 2024	KKIA	Escalator	North	31	25%	Spring	Negative

Notes: n = number of constituent swabs per pool. °C = outdoor ambient temperature at time of collection. RH = relative humidity (converted from proportion to percentage). Results show pathogen(s) detected on QIAstat-Dx Respiratory SARS-CoV-2 Panel and/or Gastrointestinal Panel 2; 'Negative' = no detection on either panel. Additional environmental indices (UV index, pollen, AQI, wind speed) and individual-swab timestamps are archived in the complete digital dataset.
